# Substantial Limitations of Ocean Alkalinity Enhancement in Mitigating the Negative Impacts of Ocean Acidification on Marine Calcifiers

**DOI:** 10.1021/acs.est.5c09298

**Published:** 2025-12-28

**Authors:** Hanna van de Mortel, Nina Bednaršek, Greg Pelletier, Richard A. Feely, Jens D. Müller, Nicolas Gruber

**Affiliations:** † HvdMortel Consulting, Utrecht 3515GS, Netherlands; ‡ Institute for Marine and Antarctic Studies, 3925 University of Tasmania, Hobart, TAS 7004, Australia; § Jožef Stefan Institute, Environmental Department, Ljubljana 1000, Slovenia; ∥ Washington Department of Ecology, 300 Desmond Dr SE, Lacey, Washington 98503, United States; ⊥ Pacific Marine Environmental Laboratory, NOAA, 7600 Sand Point Way NE, Seattle, Washington 98037, United States; # Environmental Physics, Institute of Biogeochemistry and Pollutant Dynamics, ETH Zurich, Zurich 8092, Switzerland; ∇ Carbon to Sea Initiative, 1828 L St NW, Suite 300-C, Washington, District of Columbia 20036, United States

**Keywords:** ocean alkalinity enhancement, carbon dioxide removal, ocean acidification, calcification, carbonate chemistry

## Abstract

Ocean Alkalinity Enhancement (OAE) is increasingly considered as a marine carbon dioxide removal (mCDR) strategy with the potential cobenefit of mitigating ocean acidification (OA), but this remains poorly constrained. Here, we evaluate these biological cobenefits for 27 marine calcifiers whose calcification has declined under OA, by quantifying both historical OA-driven calcification losses and the potential of OAE to reverse them under scenarios with and without air–sea equilibration. Regression models describing calcification as a function of TA-DIC reveal substantial declines since preindustrial times, particularly in linear responders (mean 22%, range 7–44%), such as gastropods and pteropods, while threshold responders show minimal decline (∼3%). A realistic addition of 50 μmol kg^–1^ of OAE alkalinity restores species-specific calcification rates maximally only between 0 and 52.2%, with the largest benefits in OA-sensitive taxa. However, restoring preindustrial calcification requires far larger TA additions (mean 104 ± 58 μmol kg^–1^ without equilibration and more than triple this amount when equilibration with atmospheric CO_2_ is considered). While higher CDR efficiency enhances atmospheric CO_2_ drawdown, it simultaneously reduces the potential for biological OA mitigation. Thus, restoration of marine calcifiers through the OAE will not necessarily align with its climate goals, complicating its application in ocean management and CDR policy.

## Graphical abstract

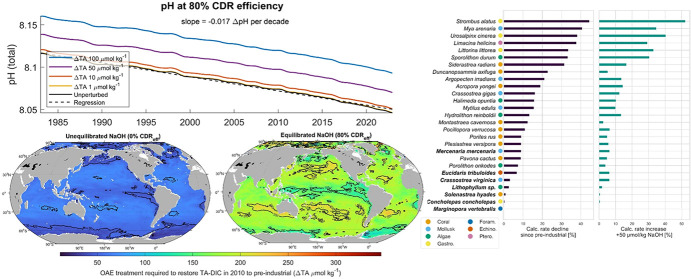

## 1 Introduction

Anthropogenic emissions have led to a significant increase in atmospheric carbon dioxide levels, rising from a preindustrial concentration of 278 ppm to over 420 ppm today.^1^ This rise poses threats to the planet’s ecosystems and biodiversity. The ocean acts as a carbon sink, absorbing roughly one-quarter of these anthropogenic CO_2_ emissions.^2,3^ This uptake of CO_2_ leads to ocean acidification (OA), which includes a decline in the carbonate ion concentration, shoaling of the saturation horizon of calcium carbonate minerals, and reduced pH.^4,5^ OA has negative effects on marine ecosystems and organisms, particularly marine calcifiers, with some of these detrimental effects already emerging under current conditions.^6,7^ OA has also been considered a planetary boundary, with its current progression approaching or transgressing the threshold.^8^

To mitigate the negative effects of climate change and limit global warming below 1.5° or 2 °C, carbon dioxide removal (CDR), including marine carbon dioxide removal (mCDR), is essential to stabilize and eventually decline atmospheric CO_2_ levels.^9^ One promising mCDR solution is ocean alkalinity enhancement (OAE). OAE enhances the ocean’s natural ability to absorb and neutralize CO_2_ from the atmosphere, by adding alkalinity (TA, eq 2) to the ocean. This alkalinity can be increased by adding alkaline substances, such as sodium hydroxide (NaOH) or sodium carbonate (Na_2_CO_3_), to the ocean (Figure S1 shows how they differentially affect carbonate chemistry). The additional hydroxide (OH^–^) or carbonate (CO_3_^2–^) ions neutralize hydrogen ions (H^+^) and increase the pH of seawater, shifting the carbonate chemistry equilibrium reactions (eq 1) toward bicarbonate (HCO_3_^–^) and CO_3_^2–^ ions. This lowers the partial pressure of CO_2_ (pCO_2_) in seawater, enabling additional uptake of atmospheric CO_2_ ^10^ leading to an increase in dissolved inorganic carbon (DIC, eq 3).

1 $${\mathrm{CO}}_{2}+H_{2}O↔H^{+}+{{\mathrm{HCO}}_{3}}^{-}↔2H^{+}+{{\mathrm{CO}}_{3}}^{2-}$$

2 $$\mathrm{TA}=\left[{{\mathrm{HCO}}_{3}}^{-}\right]+2\left[{{\mathrm{CO}}_{3}}^{2-}\right]+\left[{\mathrm{OH}}^{-}\right]-\left[H^{+}\right]+...$$

3 $$\mathrm{DIC}=\left[{\mathrm{CO}}_{2}\right]+\left[H_{2}{\mathrm{CO}}_{3}\right]+\left[{{\mathrm{HCO}}_{3}}^{-}\right]+\left[{{\mathrm{CO}}_{3}}^{2-}\right]$$

OAE has strong potential for carbon sequestration, with modeling studies highlighting its scalability and durability as a long-term CDR solution.^11–13^ In addition to carbon sequestration, OAE may offer a cobenefit by temporarily mitigating the negative OA effects on marine biota.^14–16^ Initial H^+^ neutralization through OAE increases ocean pH and [CO_3_^2–^].^17,18^ However, as CO_2_ uptake from the atmosphere progresses, these benefits diminish once atmospheric CO_2_ re-equilibrates with surface waters. In fully effective OAE, when uptake of additional CO_2_ returns the seawater pCO_2_ to the pre-enhancement level, pH and carbonate ion concentrations nearly revert to pre-TA levels, with precise changes indicated in Figure 1.

**Figure 1:**
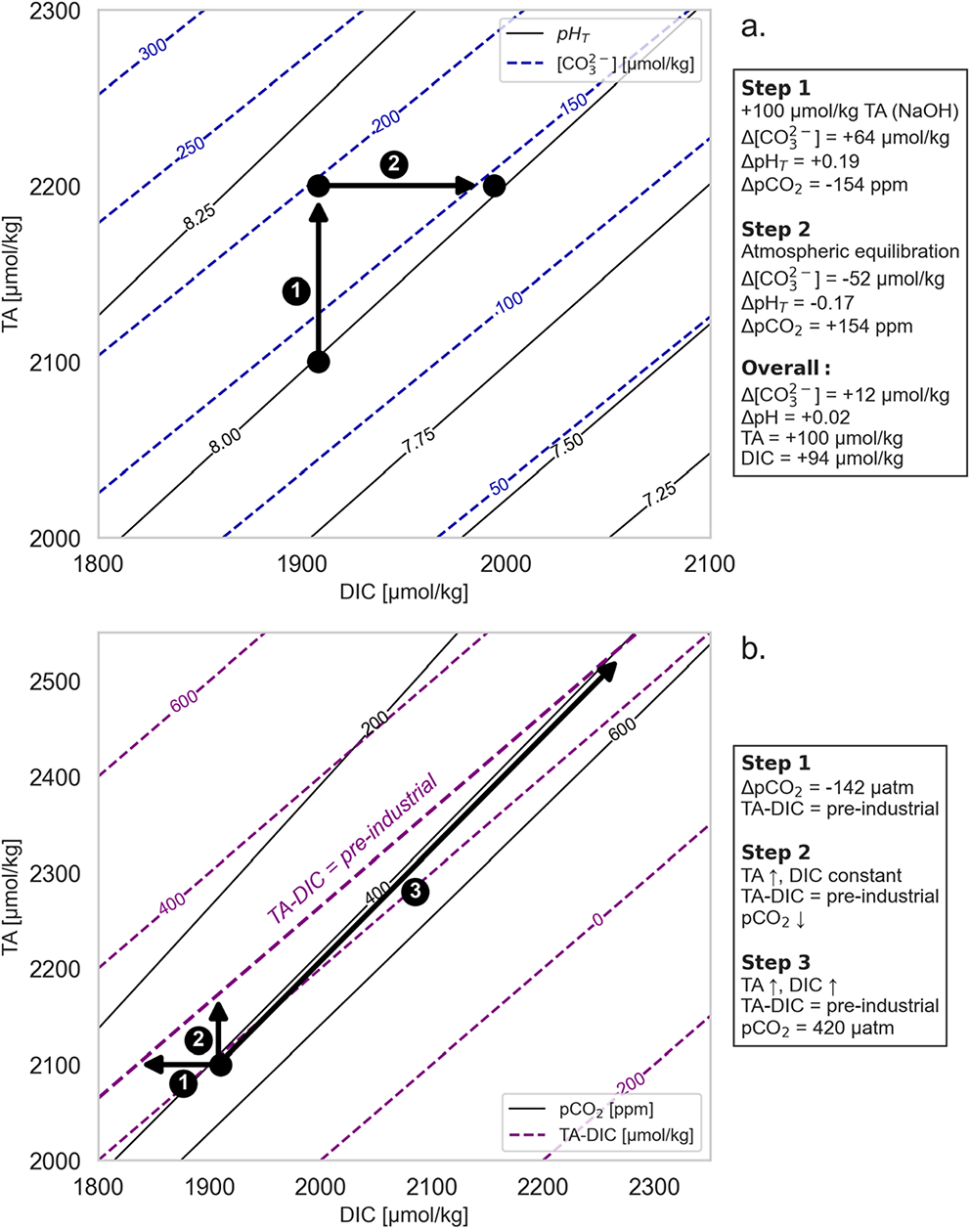
Changes in seawater–carbonate chemistry due to OAE and subsequent uptake of atmospheric CO_2_ returning pCO_2_ to the pretreatment level. Conceptual diagrams illustrating (a) the effect of OAE on pH and [CO_3_^2–^], considering a two-step procedure in which first TA is enhanced under otherwise isochemical conditions (DIC = const., Step 1) followed by a re-equilibration of the seawater sample with current atmospheric pCO_2_ (Step 2); and (b) the restoration of preindustrial TA-DIC levels, which requires to first (Step 1) approximate the preindustrial TA-DIC based on the change in atmospheric pCO_2_ over the industrial era and assuming constant TA, and then distinguishes two cases where the preindustrial TA-DIC are restored from current conditions by (Step 2) increasing only TA under constant DIC and (Step 3) increasing TA and DIC under constant current pCO_2_ levels. Calculations were done in CO2SYS,^19^ using S = 34.68, *T* = 16 °C, [Si­(OH)_4_] = 50 μmol kg^–1^, [PO_4_^3–^] = 0.5 μmol kg^–1^, and the stoichiometric dissociation constants for carbonic acid from Lueker et al.,^20^ for sulfuric acid from Dickson et al.^21^ and for total boron from Lee et al.^22^

Figure 1a illustrates this OA mitigation by conceptually dividing the OAE intervention into two steps. In Step 1 → 2, TA addition (in the form of NaOH) first increases [CO_3_^2–^] and pH, while lowering pCO_2_. In Step 2 → 3, atmospheric uptake of CO_2_ elevates pCO_2_ to the pretreatment level and decreases [CO_3_^2–^] and pH back down toward the pretreatment level. Figure 1a demonstrates how atmospheric equilibration, initial [CO_3_^2–^], and pH increases are largely reversed, despite a net increase in TA and DIC.

Model simulations in emission-driven mode show that substantial atmospheric CO_2_ reductions via OAE can prevent further OA decline.^12,27^ Despite OA mitigation potential, there are limitations under real-world scenarios due to increased CO_2_ uptake after atmospheric equilibration.^77–79^ Hauck et al.^79^ showed that OAE can initially increase surface ocean pH and enhance CO_2_ uptake, but that these effects are limited in the long term after air-sea CO_2_ equilibration occurs. Similarly, Renforth and Kruger^80^ suggest that when distributed globally in equilibrated surface waters, OAE may not be significant enough to warrant large-scale implementation, while Ilyina et al.^13^ concluded that only large-scale TA additions can boost oceanic CO_2_ uptake sufficiently to avoid further OA on the global scale– though their analysis incorporates atmospheric CO_2_ drawdown. Overall, effective OA mitigation through OAE appears feasible mainly in localized near-field settings or under extremely large TA additions.^13,26^ Overall, further research is required to understand both short- and long-term impacts of OAE intervention on marine ecosystems with respect to the mitigation potential of OA.^16,28^

Biological impacts of OAE on OA mitigation potential can be assessed by evaluating calcification rates of marine calcifiers, which are ecologically and economically important and highly abundant in coastal systems and biodiverse. Organisms’ calcification responses to OA have been extensively studied, showing widespread declines in calcification rates. OA reduces carbonate ion concentrations, lowers pH, and increases pCO_2_, accelerating calcium carbonate (CaCO_3_) dissolution and causing physiological stress. Calcification rates decrease, leading to reduced growth and development, weaker calcareous structures, and, due to energetic trade-offs, ultimately reduced survival.^29,30^ These vulnerabilities have led to marine calcifiers being recognized as indicators of ecological risks associated with OA and broader environmental changes.^31,32^ Over longer time scales, this raises concerns about biodiversity restructuring, ecological shifts of winners and losers, ecological thresholds, and potential disruptions to trophic level interactions.^7,33,34^

Calcification responses to OA vary across taxa, functional groups, and life stages.^35,36^ One key factor influencing sensitivity is the organism’s calcification mechanism, related to the use of carbonate (e.g., corals, pteropods, crustose coralline algae, some bivalves), bicarbonate ions (e.g., coccolithophores, foraminifera), or both, combined with strategies aimed at facilitating calcification.^35,37,38^ Coral reefs have experienced a 15–30% decline in skeletal density, reducing structural integrity and increasing erosion and storm damage risk.^39,40^ Mollusks show reduced calcification due to limited ion regulation, impairing carbonate balance.^34,41^ Echinoderms and crustaceans generally exhibit greater resilience as adults but display heightened sensitivity during early development.^31,35^

Similarly, species’ calcification responses to the OAE also vary. Bednaršek et al.^42^ grouped species’ responses into positive (increased calcification), neutral (no significant change) or negative (decreased calcification), identifying broad functional patterns. The study predicted that 34.4% of calcifiers responded positively, 26.0% negatively, and 39.2% neutrally. While differences were due to species-specific variability, some functional group patterns emerged. For example, corals showed largely positive and neutral responses, while dinoflagellates, calcifying algae, and pteropods were more negatively affected. While the study comparatively evaluated calcification rate change due to the OAE, it did not quantify OAE’s ability of the OAE to restore calcification lost due to the OA.

In this study, we use a subset of data from experimental OA studies,^42^ focusing on 27 marine calcifying species across a wide range of functional calcifying groups that showed a decline of calcification rates under OA, and are therefore expected to benefit from OAE. We systematically assess how calcification responses vary across seven functional groups based on their calcification mechanisms and sensitivity to carbonate chemistry, using a species-specific best-fit regression model of TA-DIC versus calcification rate. For each species, we define current and preindustrial carbonate chemistry conditions to evaluate the extent of calcification rate decline attributable to OA, with the latter based on the assumption that pCO_2_ was 142 ppm lower in preindustrial times than today. We then quantify each species’ responsiveness to the OAE treatment by calculating the change in calcification rates following the addition of a defined concentration of TA, under both unequilibrated and equilibrated air-sea exchange scenarios. Furthermore, we determine the amount of TA addition required to fully restore preindustrial calcification rates, whereby a conceptual diagram (Figure 1b) illustrates how the preindustrial conditions, in terms of our target variable TA-DIC, can be restored by OAE with and without atmospheric equilibration. Finally, we calculate the mitigation time for which a proposed TA addition restores chemical conditions or calcification rates from current conditions backward in time toward preindustrial conditions using a global monthly time-series reconstruction of surface ocean chemistry. By integrating both chemical and biological data, we aim to advance the understanding of the OAE as a strategy to mitigate OA effects across sensitive marine calcifiers that have been affected by OA.

## 2 Methods

### 2.1 Sensitivity of Marine Calcifiers to Changes in Carbonate Chemistry

To evaluate the potential of the OAE to restore calcification rates of marine species, we used published calcification rate data from experimental OA studies and developed a conceptual framework where TA is added to current baseline conditions. This method enables us to quantify species-specific responses of calcification rates to changes in the carbonate system and estimate the extent to which OAE could mitigate OA-driven declines in calcification.

We applied species-specific regression models derived from experimental OA calcification data,^42^ whereby we used TA-DIC as the independent variable. TA-DIC, also referred to as ‘Alk*’ in other studies,^43^ explains over 99% of the variability in [CO_3_^2–^] and aragonite saturation states (Ω_ar_), which are directly related to calcification (Figure S2). It is also closely related to the TA:DIC variable, used in a similar analysis by Bednaršek et al.^42^ We selected the TA-DIC variable as it allows for the most direct assessment of changes in the marine carbonate system upon changes in DIC, TA or both.

We fitted two types of regression models to the experimental data of each species to group them into two response types: (i) linear, where calcification rates increase proportionally with TA-DIC, and (ii) threshold (exponential regression), where calcification rates remain constant before dropping when a critical TA-DIC value is reached. Note that this study examines only species with a positive correlation between the calcification rate and TA-DIC. Figure 2 shows both response types with the experimental data and respective regression models shown alongside their prediction intervals. We applied an ordinary least-squares (OLS) regression model,^44^ selecting the model with the lowest p-value, considering only models with *p* < 0.05 as significant.

**Figure 2:**
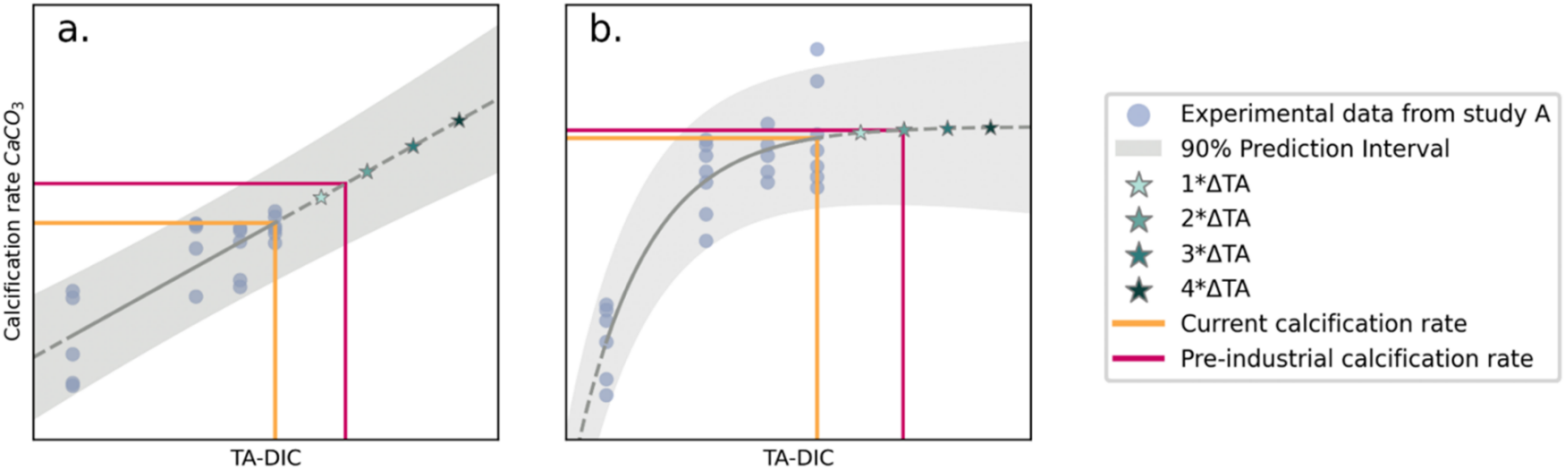
Conceptual diagrams of two positive calcification response types to OAE additions, resulting in increased TA-DIC. (a) Linear positive responder (linear calcification rate increase with increased TA-DIC) and (b) a threshold positive responder (calcification rate decline only at low TA-DIC, plateauing at higher TA-DIC). Responses were only considered linear or threshold positive when significant (*p* < 0.05). The orange line represents the current conditions and corresponding calcification rate, and the solid pink line represents the preindustrial conditions and corresponding calcification rate. The scattered dots show experimental data points, and the stars show the stepwise addition of TA from the current conditions baseline. Gray shading represents the 90% prediction interval.

### 2.2 OA Data Compilation

The experimental data used in this study consist of existing studies on marine species calcification response that aligned calcification rate data along with carbonate chemistry, and were previously compiled by Bednaršek et al.^42^ Their compiled data set covered a wide range of calcifying organisms across various functional groups and 84 species. We examined 27 species that inhabit surface waters and have shown positive responses to the OAE, with experimental control concentrations of TA and DIC deemed representative of their natural environmental conditions. Of these 27 species, 20 are linear, and 7 are threshold responders. They cover a wide range of functional groups: calcifying algae, corals, crustaceans, echinoderms, foraminifera, gastropods, pteropods, and (other) mollusks (Figure 3). Here, “gastropods” includes all taxa outside the pteropod subgroup. Most of the functional groups investigated here use carbonate ions for calcification, making it directly comparable to the TA-DIC (which approximates carbonate ion concentration; Figure S2), with only a few exceptions for the phytoplankton autotrophs that also use bicarbonate ions.

**Figure 3:**
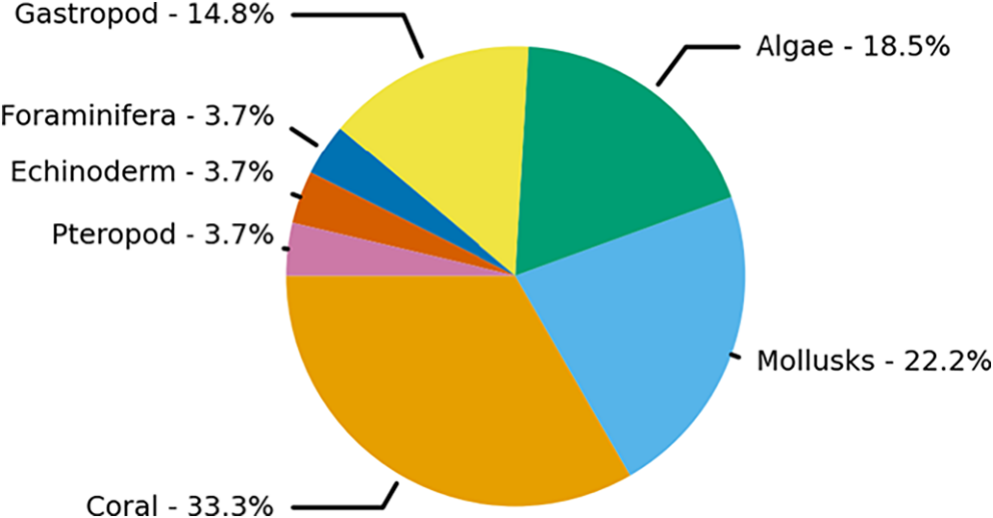
Functional group distribution among the studied marine calcifiers. Pie chart representing the relative distribution of 27 marine calcifying species across seven functional groups, including calcifying algae, corals, crustaceans, echinoderms, foraminifera, mollusks, and within, gastropods and pteropods.

A variety of calcification rate units were used across different studies, and these were standardized where possible. This standardization is thoroughly explained by Bednaršek et al.^42^ For single-cell organisms, growth rates and PIC production rates were used as indicators of the calcification rate. For some species, direct calcification rates were not reported in the literature, and only relevant parameters related to calcification (shell length, density, thickness) over time were available. While this data was also collected by Bednaršek et al.,^42^ it was not used in this study. Where there were multiple studies available for the calcification rate of one species using the same rate units, the data were combined. Data were analyzed on a species level wherever rate units were the same. TA-DIC on the *x*-axis strongly correlates to [CO_3_^2–^] (Figure S2a).

### 2.3 Computation of the Preindustrial State of Seawater–Carbonate Chemistry

We define a current conditions baseline based on species-specific experimental control TA, DIC, temperature, and salinity. We assume experimental control conditions to be representative of natural conditions in each species’ typical habitat. From TA and DIC, we derived the current mean pCO_2_ values of the species’ natural conditions. To estimate preindustrial conditions, we assumed pCO_2_ was 142 ppm lower than today, based on the difference between the global current atmospheric pCO_2_ of 420 ppm^1^ and preindustrial pCO_2_ of 278 ppm,^45^ reflecting that—in the long term—the growth in seawater pCO_2_ tends to closely follow the growth in the atmospheric pCO_2_, while maintaining the regional air-sea disequilibrium. This methodology is visualized in Step 1 of Figure 1b, where the origin of the three arrows represents the current conditions. We assume TA has not changed since the preindustrial era, which is supported by only minor observed changes in TA over the past 40 years,^46,47^ and by model studies indicating the stability of TA over the industrial era.^48^ Since we assume TA has remained constant since the preindustrial era, the change in TA-DIC is equal to the change in DIC. Likewise, the experimental control temperature and salinity were used to compute the preindustrial conditions, i.e., long-term changes in temperature and salinity were not incorporated. Carbonate system calculations were done in CO2SYS,^19^ using [Si­(OH)_4_] = 50 μmol kg^–1^, [PO_4_^3–^] = 0.5 μmol kg^–1^, and the stoichiometric dissociation constants for carbonic acid from Lueker et al.,^20^ for sulfuric acid by Dickson et al.^21^ and for total boron from Lee et al.^22^

### 2.4 Calcification Decline Due to OA

We quantify the extent of calcification rate decline attributable to OA by estimating the relative decline in calcification rate since the preindustrial era compared to current conditions. This provides a standardized, mechanistic measure of OA-driven biological changes across species. We use the current and preindustrial calcification rates, which correspond to the current and preindustrial TA-DIC conditions, respectively, derived with each species’ regression model.

### 2.5 Equilibrium Computation of the Marine Carbonate System for OAE with and without CO_2_ Uptake from the Atmosphere

To quantify the potential of OA mitigation via an OAE (Section 2.6), we consider scenarios both with and without air-sea equilibration. To incorporate air-sea equilibration into our calculations, we quantified both the theoretical maximum OAE efficiency (ηmax) and the CDR efficiency (see below), which reflect the actual atmospheric CO_2_ removal, considering the limitation of CO_2_ uptake by the background state of seawater–carbonate chemistry and the incomplete realization of the full CDR potential due to delayed air-sea gas exchange.

The theoretical maximum OAE efficiency (ηmax) is defined by Yankovsky et al.^49^ as the maximum increase in moles of DIC that could occur through mCDR for a given increase in moles of TA through OAE. To determine the ηmax, we calculated the hypothetical change in TA-DIC that would be caused by a permanent increase of TA by 1, 10, and 100 μmol kg^–1^ under constant pCO_2_ (representing the case of full equilibration of the added TA with the atmosphere) using the OceanSODA-ETHZv2023 ^50^ gridded data set over the 2018 to 2022 period at 1° × 1° horizontal resolution. We assume that the alteration of atmospheric CO_2_ by the OAE can be neglected.

For the 26 species inhabiting the global coastal region, an area-weighted mean ηmax of 0.83 was calculated for grid cells within 300 km of the nearest coast. For the species *‘* *Limacina helicina* *’* we defined a ‘polar’ region, encompassing grid cells north of 60°N or south of 60°S, giving a mean ηmax of 0.904 (see Table S1). See Figure S4 for maps showing the 2018–2022 ηmax values in the grid cells averaged for the coastal and polar regions as well as globally. Table S2 shows the collection locations and regions per species.

With ηmax, we can also compute the CDR efficiency, which describes the effectiveness of actual atmospheric CO_2_ removal relative to ηmax.^51^ Unlike ηmax, which can be estimated from static carbonate chemistry reconstructions, the quantification of CDR efficiency requires dynamic modeling of air-sea gas exchange processes to capture the gradual equilibration of CO_2_ between the ocean and the atmosphere. Hence, we adopted CDR efficiency estimates from the literature for our offline computations (see below). Full methodological details, including equations and computational procedures, are provided in Supplementary Text S1.

Recent studies indicate CDR efficiency to be location and time dependent, with lower values ranging between 0.2 and 0.85 over the first few years of continuous OAE loading, and between 0.65 and >0.95 afterward^26,51,52^ or with the estimates on the global scale of 0.71–0.84.^53^ As such, we have chosen CDR efficiency to be 80% for our analysis, which aligns well with the estimates by Yamamoto et al.^54^

### 2.6 Quantifying OA Mitigation via OAE

To evaluate the biological and chemical implications of TA addition, we have considered three different approaches to determine OAE benefits in terms of calcification rate increase. First, we calculated the amount of TA required to restore calcification back to the preindustrial conditions. Second, we determined a species-specific calcification increase at the addition of 50 μmol kg^–1^. For both of these approaches, we considered four scenarios of TA addition that distinguish the source of TA added (NaOH or Na_2_CO_3_) and whether CO_2_ uptake from the atmosphere was realized or not (with or without equilibration with the atmosphere). The third approach determines the OAE mitigation of the OAE, i.e., the temporal recovery from OA for a given alkalinity enhancement. This was only done for two scenarios, involving equilibration with the atmosphere for both NaOH and Na_2_CO_3_.

#### 2.6.1 TA additions Required to Restore Preindustrial Conditions

For our first approach, we computed the required amount of TA added to restore preindustrial TA-DIC (Step 2 or 3 of Figure 1b). Note that the amount of TA required to restore preindustrial conditions is not biologically controlled; it is solely dependent on the experimental TA, salinity, and temperature. Variability in experimental conditions (temperature, salinity) contributes to uncertainty in these estimates, as shown in Table S3. While this actual concentration of TA required is shown in Tables 1 and S4, in our figures, we have added four increments of TA (ΔTA; in steps of 50 μmol kg^–1^) from the current conditions baseline, assuming unequilibrated conditions (see Figure 2). This is solely to visualize this conceptual addition of TA and give a visual indication of the magnitude of TA addition to restore preindustrial conditions.

**Table 1:** Summary of OA Study Data, Changes in the Calcification Rates, and Biological Responses to the Calcification of the Phosphorus of OAE^a^

Experimental data			Best-fit regression		Current Conditions		Preindustrial conditions		OA	OAE needed (to restore PI conditions)		OAE (calc. increase +50 μmol kg^–1^ NaOH)	
Studies	Group	Species	Rate unit	Response	TA-DIC [μmol kg^–1^]	Calcification rate	TA-DIC [μmol kg^_1^]	Calcification rate	Calc. drop since PI [%]	ΔNaOH, no eq [μmol kg^–1^]	ΔNaOH, with eq [μmol kg^–1^]	No eq [%]	With eq [%]
^87^	Algae	*Halimeda opuntia*	mmol/m^2^/h	*lin.*	292	8.32 × 10^–02^ ± 4.16 × 10^–02^	384	9.88 × 10^–02^ ± 4.44 × 10^–02^	15.82	91	273	10.28	3.44
 ^88^	Algae	*Hydrolithon reinboldii*	mmol/g/h	*lin.*	243	2.79 × 10^–03^ ± 4.40 × 10^–03^	300	3.22 × 10^–03^ ± 4.40 × 10^–03^	13.31	57	172	13.39	4.48
 ^89^	Algae	*Lithophyllum sp.*	mmol/g/h	*thresh.*	293	2.34 × 10^–01^ ± 1.87 × 10^–01^	386	2.41 × 10^–01^ ± 1.88 × 10^–01^	2.68	93	278	1.87	0.76
 ^90^	Algae	*Porolithon onkodes*	mmol/m^2^/h	*lin.*	333	2.16 × 10^00^ ± 1.34 × 10^00^	437	2.33 × 10^00^ ± 1.35 × 10^00^	7.42	104	312	3.85	1.29
 ^91,92^	Algae	*Sporolithon durum*	mmol/m^2^/h	*lin.*	259	1.48 × 10^–01^ ± 3.00 × 10^–01^	340	2.21 × 10^–01^ ± 3.05 × 10^–01^	33.19	81	244	30.48	10.19
 ^91,92^	Coral	*Acropora yongei*	mmol/m^2^/h	*lin.*	259	4.92 × 10^00^ ± 3.32 × 10^00^	340	6.08 × 10^00^ ± 3.36 × 10^00^	19.02	81	244	14.41	4.82
 ^58^	Coral	*Duncanopsammia axifuga*	mmol/m^2^/h	*lin.*	486	2.39 × 10^01^ ± 9.27 × 10^00^	765	3.11 × 10^01^ ± 1.04 × 10^01^	22.90	279	835	5.32	1.78
 ^58^	Coral	*Montastraea cavernosa*	mmol/m^2^/h	*lin.*	486	2.17 × 10^00^ ± 8.67 × 10^–01^	765	2.48 × 10^00^ ± 9.12 × 10^–01^	12.46	279	835	2.55	0.85
 ^88^	Coral	*Pavona cactus*	mmol/m^2^/h	*lin.*	328	3.64 × 10^00^ ± 1.59 × 10^00^	428	3.99 × 10^00^ ± 1.61 × 10^00^	8.86	100	299	4.86	1.63
 ^92^	Coral	*Plesiastrea versipora*	mmol/m^2^/h	*lin.*	270	2.32 × 10^00^ ± 1.05 × 10^00^	357	2.55 × 10^00^ ± 1.07 × 10^00^	9.02	87	259	5.72	1.91
 ^94^	Coral	*Pocillopora verrucosa*	mmol/m^2^/h	*lin.*	321	2.49 × 10^00^ ± 1.43 × 10^00^	417	2.79 × 10^00^ ± 1.47 × 10^00^	10.99	96	287	6.44	2.15
 ^88^	Coral	*Porites rus*	mmol/m^2^/h	*lin.*	328	6.31 × 10^00^ ± 3.47 × 10^00^	428	6.94 × 10^00^ ± 3.51 × 10^00^	9.04	100	298	4.98	1.67
 ^83^	Coral	*Siderastrea radians*	mmol/m^2^/h	*lin.*	390	5.08 × 10^00^ ± 4.72 × 10^00^	529	7.40 × 10^00^ ± 4.89 × 10^00^	31.45	138	413	16.60	5.55
 ^83^	Coral	*Solenastrea hyades*	mmol/m^2^/h	*thresh.*	395	2.81 × 10^00^ ± 3.52 × 10^00^	537	2.83 × 10^00^ ± 3.64 × 10^00^	0.97	141	423	0.62	0.27
 ^96^	Echino.	*Eucidaris tribuloides*	mmol/g/h	*thresh.*	181	5.68 × 10^–04^ ± 7.33 × 10^–04^	244	6.09 × 10^–04^ ± 7.70 × 10^–04^	6.75	63	188	6.28	2.68
 ^98^	Foram.	*Marginopora vertebralis*	mmol/g/h	*thresh.*	405	1.90 × 10^–03^ ± 6.94 × 10^–04^	561	1.90 × 10^–03^ ± 7.23 × 10^–04^	0.00	156	466	0.00	0.00
 ^82^	Gastro.	*Concholepas concholepas*	mmol/g/h	*thresh.*	107	3.37 × 10^–03^ ± 1.57 × 10^–03^	141	3.38 × 10^–03^ ± 1.59 × 10^–03^	0.43	34	103	0.49	0.29
 ^96^	Gastro.	*Littorina littorea*	mmol/g/h	*lin.*	202	2.20 × 10^–04^ ± 3.06 × 10^–04^	278	3.32 × 10^–04^ ± 3.15 × 10^–04^	33.53	76	229	33.00	11.04
 ^96^	Gastro.	*Strombus alatus*	mmol/g/h	*lin.*	202	1.49 × 10^–04^ ± 1.76 × 10^–04^	278	2.67 × 10^–04^ ± 1.86 × 10^–04^	44.37	76	229	52.17	17.45
 ^96^	Gastro.	*Urosalpinx cinerea*	mmol/g/h	*lin.*	202	1.50 × 10^–04^ ± 1.58 × 10^–04^	278	2.42 × 10^–04^ ± 1.64 × 10^–04^	38.16	76	229	40.37	13.50
 ^96^	Mollusk	*Argopecten irradians*	mmol/g/h	*lin.*	235	4.93 × 10^–04^ ± 3.32 × 10^–04^	334	6.24 × 10^–04^ ± 3.62 × 10^–04^	21.09	99	296	13.51	4.52
 ^99^	Mollusk	*Crassostrea gigas*	mmol/g/h	*lin.*	246	3.03 × 10^–04^ ± 7.38 × 10^–05^	324	3.61 × 10^–04^ ± 8.29 × 10^–05^	16.14	78	233	12.34	4.13
 ^81,96^	Mollusk	*Crassostrea virginica*	mmol/g/h	*thresh.*	98	2.22 × 10^–04^ ± 5.08 × 10^–04^	120	2.29 × 10^–04^ ± 5.10 × 10^–04^	3.22	22	67	6.30	2.58
 ^96^	Mollusk	*Mercenaria mercenaria*	mmol/g/h	*thresh.*	235	6.38 × 10^–05^ ± 5.50 × 10^–05^	334	7.01 × 10^–05^ ± 5.88 × 10^–05^	8.96	99	296	6.39	2.58
 ^96^	Mollusk	*Mya arenaria*	mmol/g/h	*lin.*	235	1.03 × 10^–03^ ± 7.28 × 10^–04^	334	1.74 × 10^–03^ ± 8.23 × 10^–04^	40.70	99	296	34.71	11.61
 ^66,96^	Mollusk	*Mytilus edulis*	mmol/g/h	*lin.*	243	3.08 × 10^–04^ ± 2.96 × 10^–04^	335	3.64 × 10^–04^ ± 3.03 × 10^–04^	15.62	92	275	10.08	3.37
 ^37^	Pteropod	*Limacina helicina*	mmol/g/h	*lin.*	165	5.44 × 10^–04^ ± 1.81 × 10^–04^	268	8.73 × 10^–04^ ± 2.18 × 10^–04^	37.70	103	373	29.31	8.12

a Summary of all OA studies for linear and threshold positive responders, including references, species/group names and calcification rate units. The type of calcification response to OA for each studied species was determined from the best-fitting regression models of the calcification rate as a function of TA-DIC. Statistical metrics for each regression model can be found in Table S3, along with other additional information. TA-DIC and species-specific calcification rates (±90% prediction interval) are given for current and preindustrial conditions. The OA response is shown here by the relative decline in calcification rate from preindustrial (PI) to the current conditions. The responsiveness to the OAE is shown by two metrics: NaOH addition required to return to TA-DIC from current to preindustrial conditions, as well as the calcification rate increase upon 50 μmol kg^–1^ NaOH addition, considering both nonequilibrated and equilibrated OAE. The equilibrated OAE scenario takes into account 80% CDR efficiency with ηmax based on the species region (coastal = 0.832 and polar = 0.904).

#### 2.6.2 Calcification Rate Restoration for a Given TA Enhancement

For our second approach, we quantify species-specific biological responses to the OAE by estimating how much the calcification rate improves upon the enhancement of TA by a fixed concentration. We refer to this as ‘responsiveness to OAE’. We choose a concentration of 50 μmol kg^–1^ TA in addition to analyze this species-specific responsiveness, because such concentrations are potentially considered in the range of coastal regions’ initial alkalinity variability,^55,56^ but is large enough to instigate a noticeable calcification rate increase. We use the current and preindustrial calcification rates corresponding to the current and preindustrial TA-DIC conditions, respectively.

#### 2.6.3 Calculating time for OA Mitigation Based on TA Additions

To explore different OA mitigation scenarios under past trends and variability in the marine CO_2_ system, we applied offline computations to an observation-based reconstruction of the surface ocean chemistry. We used the global monthly time-series reconstruction of surface ocean chemistry between 1982 and 2022 at 1° × 1° horizontal resolution obtained from OceanSODA-ETHZv2023.^50^ We extracted monthly TA-DIC and computed 12-month moving average values to smooth the seasonal variations.

To estimate the effect of OAE toward chemical OA mitigation, TA was continuously added to an unperturbed line (ΔTA = 0) in each month in different treatment concentrations (ΔTA = 1, 10, or 100 μmol kg^–1^, assuming air-sea CO_2_ equilibration) over 1982–2022. To further explore the concept of OA mitigation, we quantify the time intervals (‘mitigation time’) for which a proposed TA addition restores chemical conditions or calcification rates from current conditions backward in time toward preindustrial conditions. For example, if a ΔTA value of 100 μmol kg^–1^ corresponds to a mitigation time of 10 years, this indicates that the addition of ΔTA improves the TA-DIC to levels that were present in the unperturbed scenario 10 years earlier. The ‘mitigation time’ was calculated using the following equation:

4 Mitigation time=Mean(OAE Treatment−Unperturbed)/Slope of Unperturbed

Where ‘OAE Treatment’ and ‘Unperturbed’ refer to the time series of the target variable (e.g., TA-DIC) with and without TA addition (control without perturbation), respectively.

To relate the analysis of the chemical OA mitigation potential based on OceanSODA to the assessment of calcification rate responses for the 1982–2022 period, we applied the regression coefficients in Table 1 to the time series of TA-DIC with and without OAE, to estimate the time series of calcification rates over the same period for each species. Per species, the difference in calcification rate for each ΔTA scenario (1, 10, and 100 μmol kg^–1^) was calculated, as well as the mitigation time.

### 2.7 Prediction Intervals

Prediction intervals were calculated for each regression model based on experimental data, best-fit model parameters, prediction points, and a significance level of 0.1 (yielding 90% intervals). Prediction intervals account for model uncertainty and biological variability in the OA experiments and are especially important for estimating values beyond the observed data range. This interval was computed using a two-tailed t-distribution to derive critical t-values, which define the upper and lower bounds around the predicted values. These bounds indicate the expected range of calcification responses and reflect model confidence at each prediction point and are summarized in Table 1 for current and preindustrial conditions. To support interpretation of model reliability, the residual sum of squares, residual mean square error, and standard error of the estimate are provided. These metrics quantify the unexplained variance and help evaluate the accuracy and predictive strength of the regression fit.

## 3 Results

### 3.1 Comparing Calcification Rate Responses Across Species

This study included 27 species, covering a broad range of coastal and open-water calcifying species from different habitats, providing a variety of potential responses to the OAE. Across all calcifying species, calcification rates have dropped with a range of 0.0 to 44.4%, and an average of 17.2%. This variability reflects differences in calcification mechanisms and response types (linear vs threshold). A conceptual figure (Figure 4) illustrates calcification response of three different calcifying species, which are species’ responses based on the experimental data and were added to aid in interpreting species differences to OA. Each species differs in the calcification rate slopes relative to TA-DIC, which is closely correlated to [CO_3_^2–^] and Ω_ar_ (Figure S2), as well as their current and preindustrial conditions. Species A shows a faster decline in calcification since preindustrial times and therefore responds faster and more strongly to increasing TA-DIC, as related to OAE enhancement. In comparison, the calcification rate of Species B declines more slowly and is less responsive to the TA-DIC increase. The same can be said for species D compared with species C, where species D is more responsive to the OAE than species C.

**Figure 4:**
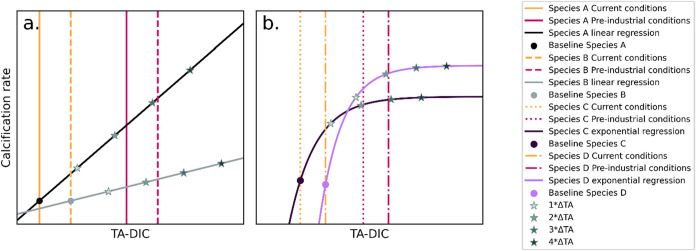
Differential sensitivity of positive calcifiers to OAE. Conceptual diagram showing differences in calcification rates (*Y*-axis) in response to changes in TA-DIC (*X*-axis) for (a) two linear responders and (b) two threshold responders. Conceptually, TA is added at different specific increments (1*ΔTA, 2*ΔTA, 3*ΔTA, and 4*ΔTA) starting from the circle symbols (‘Baseline’) to visualize the amount of TA addition required to restore preindustrial TA-DIC. This figure shows differential sensitivities to OAE, with species A experiencing a stronger recovery of calcification rates due to OAE compared to species B, and species D experiencing a stronger recovery of calcification rates compared to species C.

Current TA-DIC conditions from the experimental data control conditions range from 98 to 486 μmol kg^–1^, averaging at 276 ± 98 μmol kg^–1^, whereas preindustrial TA-DIC conditions are higher and range from 120 to 765 μmol kg^–1^, averaging at 379 ± 151 μmol kg^–1^. Given that current and preindustrial TA-DIC conditions were computed using constant TA, the change in TA-DIC is exclusively due to the increase in DIC ranging from 22 to 279 μmol kg^–1^ and averaging at 104 ± 58 μmol kg^–1^. The mean and range of ΔDIC determined from experimental conditions overlaps with the total change of anthropogenic carbon in the surface layer of the ocean over the industrial period (global mean: 60 μmol kg^–1^, range: 40–90 μmol kg^–1^).^5^ These species-specific TA-DIC conditions can be used together with the best-fit regression model to compute current and preindustrial calcification rates.

### 3.2 Reduced Calcification Rates since Preindustrial

Linear calcification responders show a significantly larger reduction of calcification, ranging from 7.4 to 44.4%, with an average of 22.5%. Comparatively, the calcification for the threshold responders to OA ranges from 0 to 9.0%, with an average of 3.3% decline since preindustrial times (see Table 1 and Figure 5).

**Figure 5:**
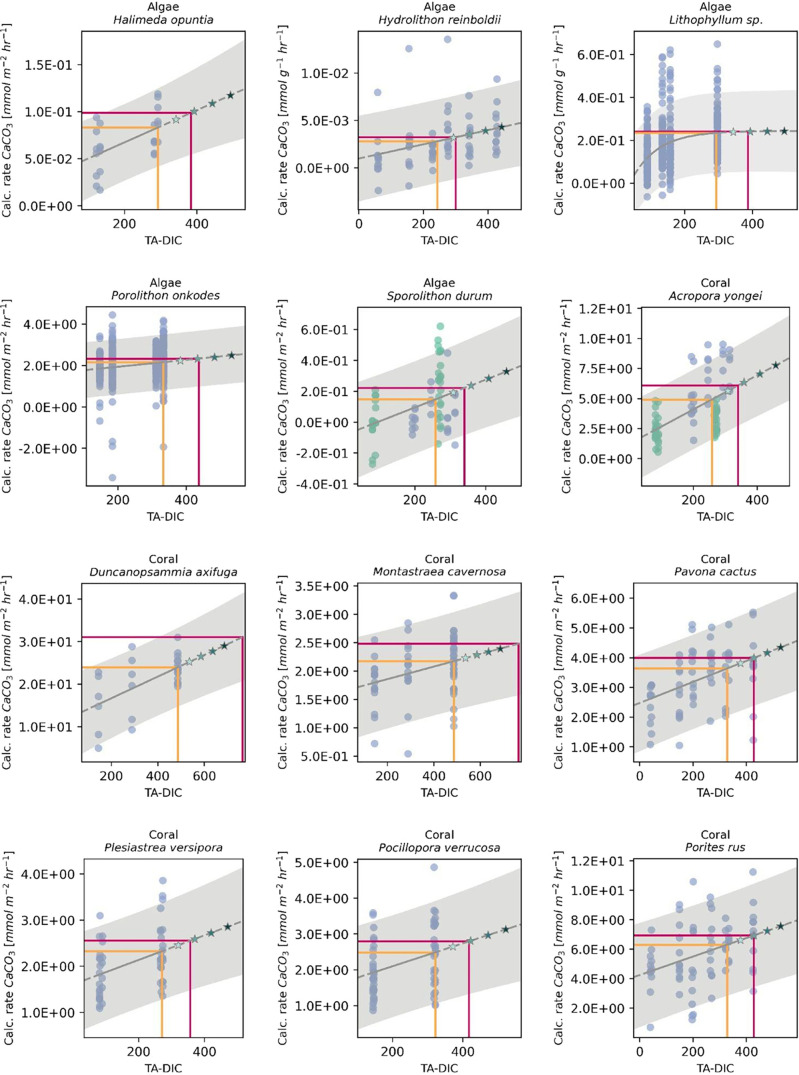

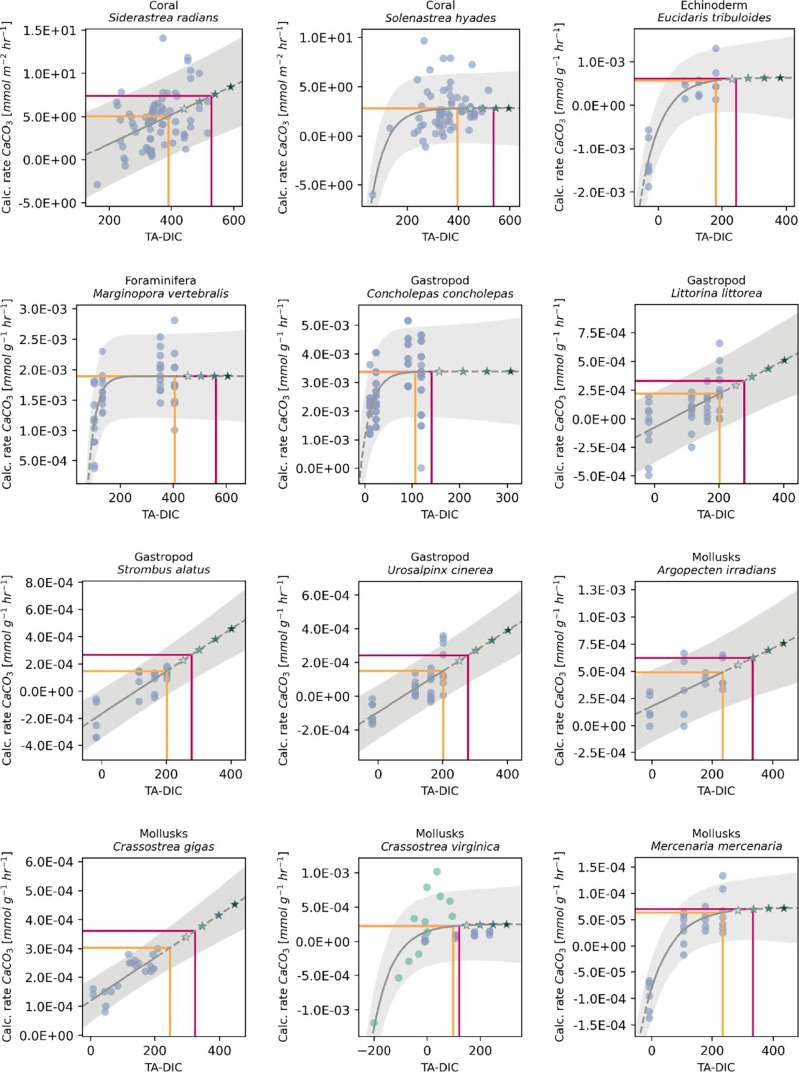

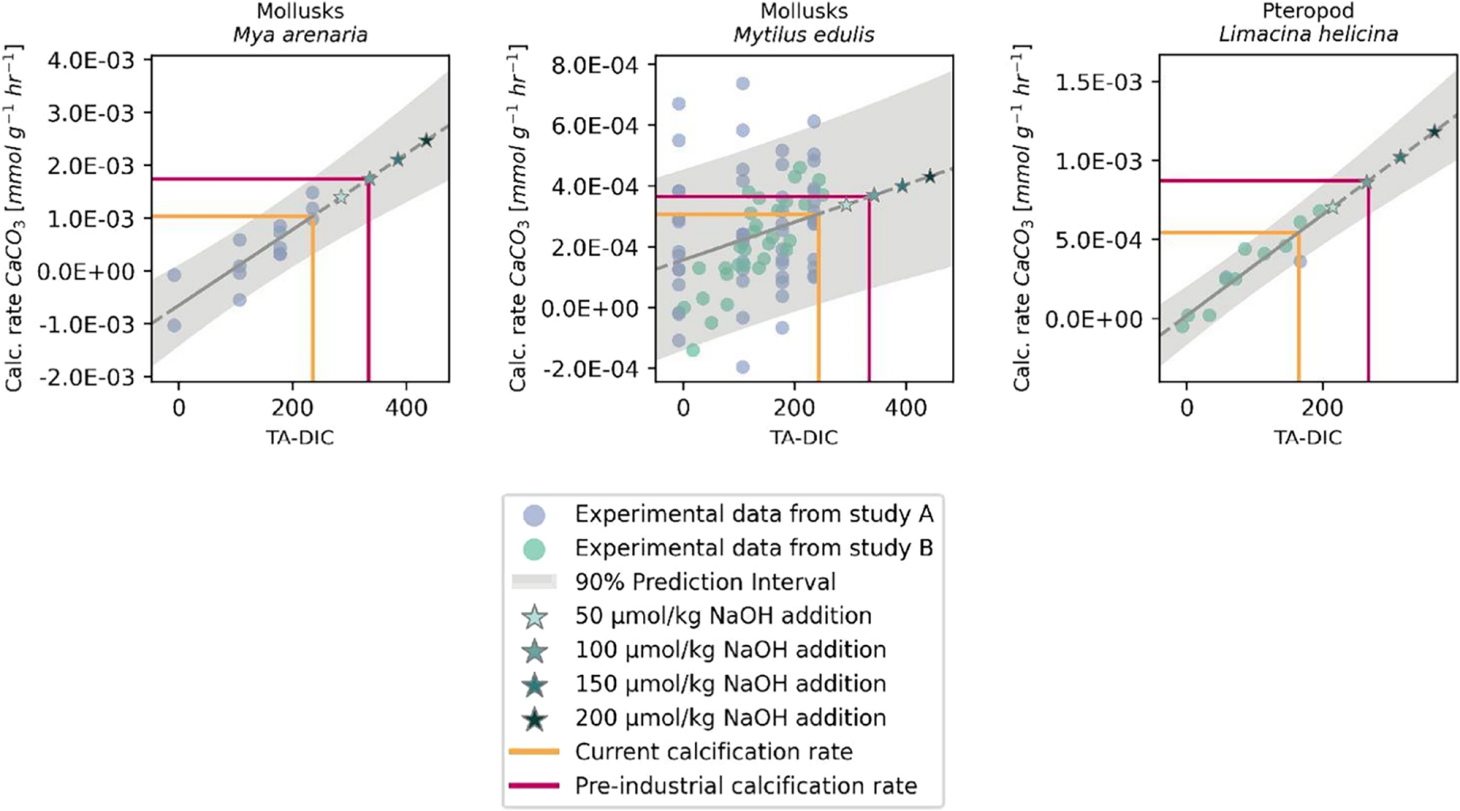
Positive responses of 30 marine calcifiers to the OAE. Changes in calcification rates as a function of TA-DIC, shown with the best-fitting regression models for each species. Responses were only considered when significant (*p* < 0.05). Circles represent the experimental data points, and the stars show the incremental addition of NaOH from the current conditions baseline in steps of 50 μmol kg^–1^ up until a total of 200 μmol kg^–1^. Current and preindustrial calcification rates and TA-DIC conditions are indicated by orange and pink lines, respectively. Gray shading represents the 90% prediction interval. Note that the predictor variable TA-DIC is strongly correlated with [CO_3_^2–^] and Ω_ar_ (Figure S2).

Out of the linear positive calcifiers, which have been most severely impacted by OA, the highest relative decline in calcification rate is observed in gastropods and pteropods, whose calcification rates have declined by an average of 38.4 ± 4.5%. The most affected species are *Strombus alatus* (gastropod), *Mya arenaria* (mollusk), *Urosalpinx arenaria* (gastropod), and *Limacina helicina* (pteropod). One coral species, *Siderastrea radians*, also shows a large decline (∼46%), while other corals (e.g., *Pavona cactus*, *Plesiastrea versipora*, *Porites rus*, *Pocillopora verrucosa*, and *Montastrea cavernosa*) and calcifying algae (*Porolithon onkodes*, *Hydrolithon reinboldii*) show relatively smaller declines.

### 3.3 The Potential of Chemical and Biological OA Mitigation via OAE

#### 3.3.1 The Slope of Calcification Rates Determines the Responsiveness to OAE

Across all species, 50 μmol kg^–1^ unequilibrated NaOH addition allows for increase of calcification rates over a range of 0.0 to 52.2%, with an average of 13.6 ± 13.8% (Table 1; see Table S4 for Na_2_CO_3_ results). For linear responders, the increase in calcification ranges between 2.6 to 52.2% and averages at 17.4 ± 14.7%, while for the threshold responders, it ranges between 0.0 and 6.4% and averages at 3.1 ± 3.0% The species that show the greatest increase in calcification rate include the gastropods *Strombus alatus* (gastropod), *Urosalpinx cinerea* (gastropod), *Mya arenaria* (mollusk), and *Littorina littorea* (gastropod). Species that will experience the lowest responsiveness to OAE treatment are all the threshold responders and linear responders *Montastrea cavernosa* (coral), *Porolithon onkodes* (calcifying algae), *Pavona cactus* (coral), *Duncanopsammia axifuga* (coral), and *Plesiastrea versipora* (coral).

#### 3.3.2 Restoring Calcification Rates to Preindustrial Conditions

To restore the calcification rate from the current to the preindustrial conditions for linear responders, 76 to 279 μmol kg^–1^ NaOH addition is required without equilibration, and with equilibration, this is 229 to 835 μmol kg^–1^ (Table 1). Species that have experienced a large decline in calcification rate due to OA (Figure 6a) are generally most responsive to OAE, as indicated by calcification rate increase upon NaOH addition by a fixed concentration of 50 μmol kg^–1^ (Figure 6b). The enhancement is influenced by the sensitivity of the calcification rate to changes in seawater–carbonate chemistry. If the OAE is achieved by Na_2_CO_3_ addition, the required equivalent TA increase amounts to 153 to 559 μmol kg^–1^ and 326 to 1191 μmol kg^–1^, without and with equilibration, respectively (Table S4).

**Figure 6:**
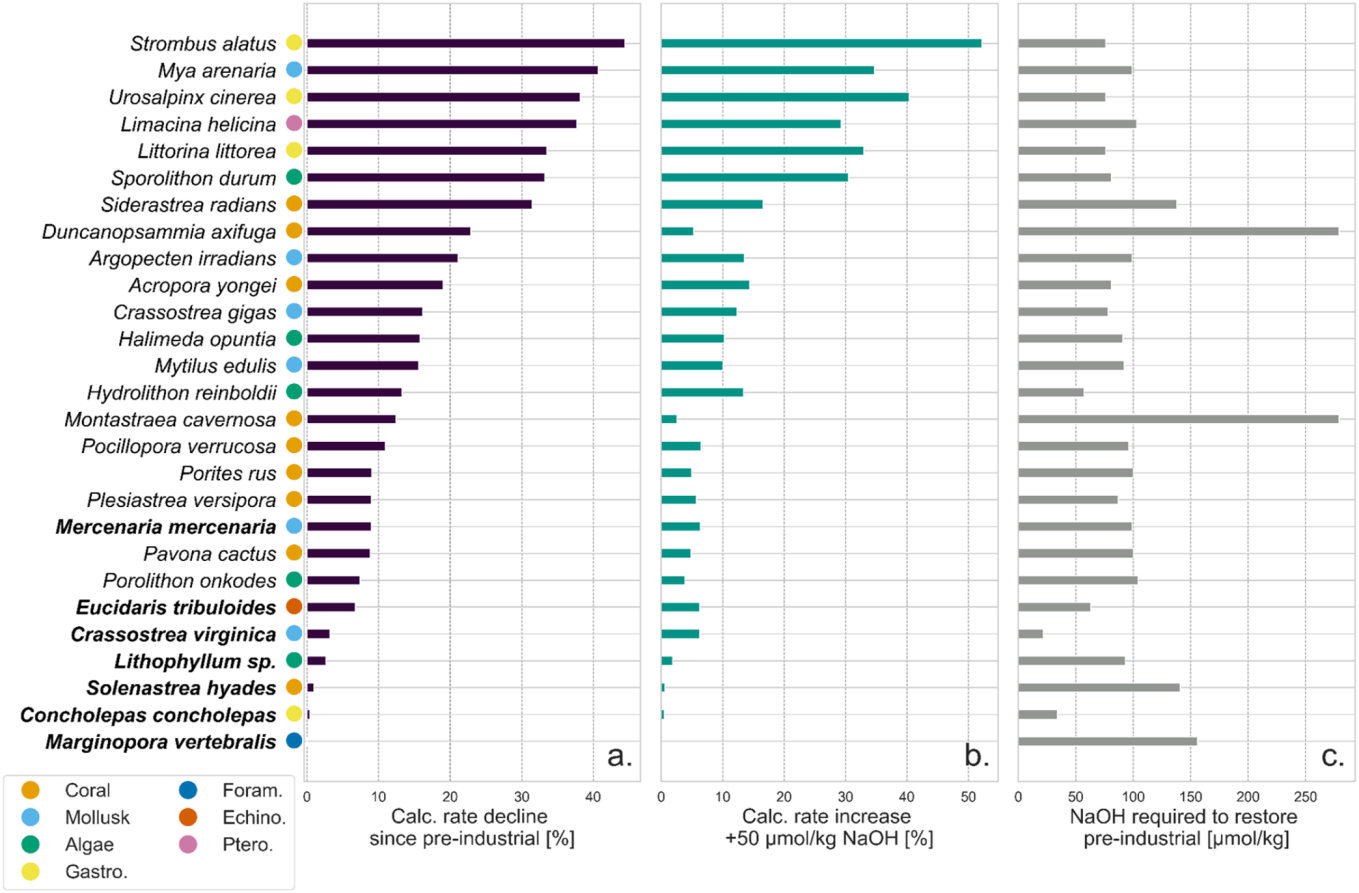
Calcification rate decline due to OA, calcification rate increase upon 50 μmol/kg NaOH addition, and NaOH addition required to restore preindustrial conditions. (a) Decline of species-specific calcification rates from preindustrial to current conditions, (b) the relative increase in calcification rate compared to the current conditions baseline upon 50 μmol kg^–1^ NaOH addition, and (c) the concentration of NaOH addition required to restore calcification to the preindustrial conditions assuming unequilibrated conditions. Bold species names indicate threshold positive responders, while the remaining species show linear responders. The colored dots indicate functional groups, which are sorted in descending order of calcification rate decline.

In contrast, the concentration of NaOH required to restore preindustrial conditions (Figure 6c) reflects the amount of TA addition needed to shift from current to preindustrial TA-DIC and, thus, is determined solely by carbonate chemistry (i.e., the control experimental conditions). Under the assumed equilibrium with atmospheric pCO_2_, the TA-DIC variable, which is a proxy for the carbonate ion concentration, is generally lower under low salinities and low temperatures (see Figure S5a,b for the correlation of the current TA-DIC against temperature and salinity; R^2^ = 0.39 for both). As a result, the amount of NaOH required to restore preindustrial conditions also varies across experiments, shown in Figure S5c by the strong linear relationship between NaOH addition and TA-DIC (R^2^ = 0.94). In general, more NaOH addition is required under high TA-DIC levels, because these waters have a higher buffer capacity and, consequently, experienced a higher absolute decline in carbonate ion concentration since the preindustrial period.

#### 3.3.3 Mitigation Time

Because of continued CO_2_ uptake in surface waters, a significant (*p* < 0.05) declining trend in TA-DIC is observed over the 1982–2022 period (Figure 7). Our estimates reveal that different concentrations of ΔTA can differentially contribute to elevated TA-DIC compared to the unperturbed conditions, depending on the CDR efficiency. We show the effect of various OAE additions of ΔTA on TA-DIC, pH, and Ω_ar_ in the coastal region with a CDR efficiency of 80% (Figure 7) and 100% (Figures S6–S8 and Table S5).

**Figure 7:**
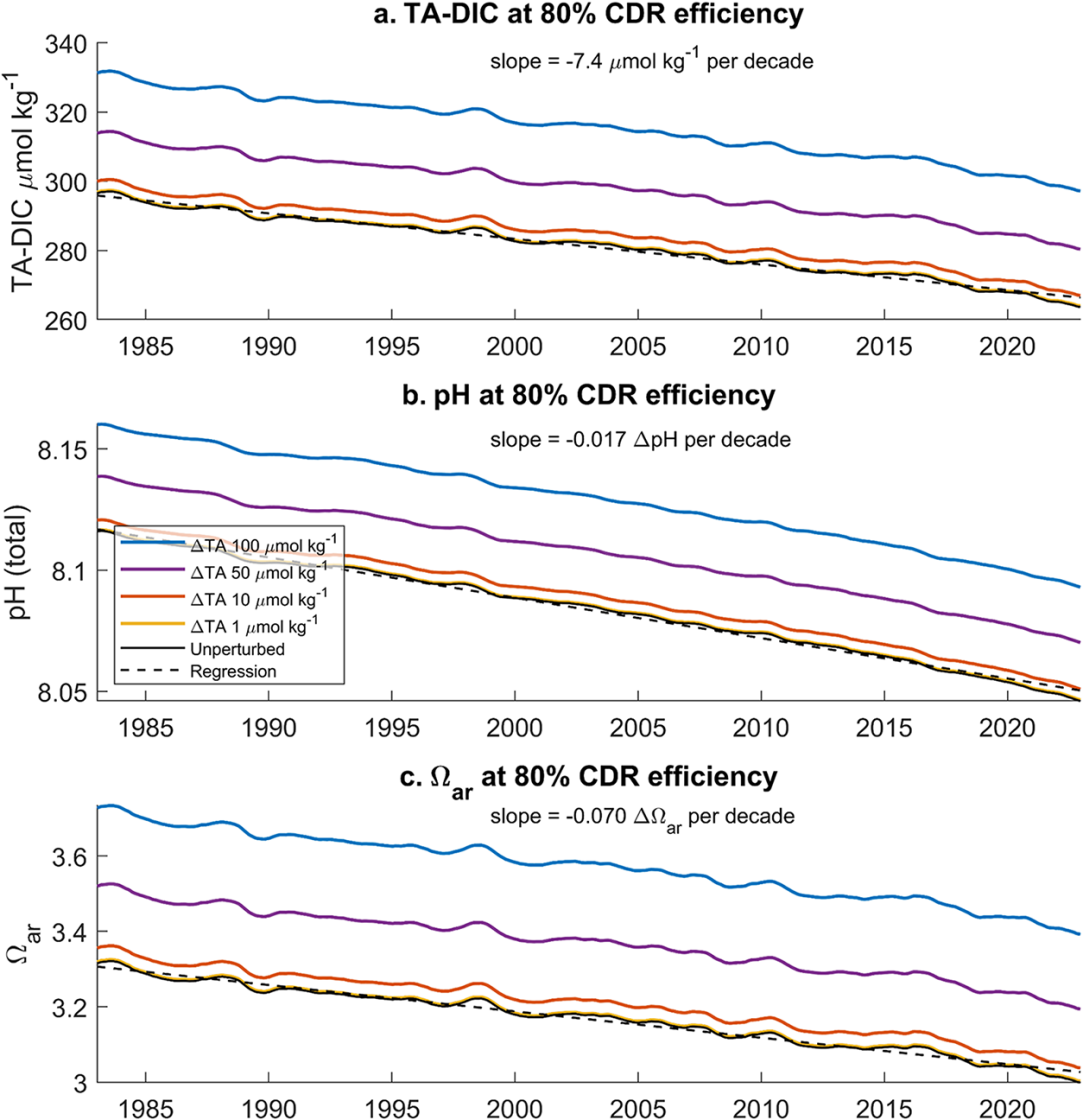
Time series for the restoration of TA-DIC, pH_T_, and Ω_ar_ for four levels of TA additions. Time series for (a) TA-DIC, (b) pH_T_, and (c) Ω_ar_ at different TA additions from 1985 to 2022 compared to the unperturbed conditions. Computations are done assuming ηmax = 0.832 (coastal region) and 80% CDR efficiency.

A ΔTA treatment of 50 μmol kg^–1^ in the coastal region restores TA-DIC, pH, and Ω_ar_ to the conditions of 23-, 14-, and 29-years prior, respectively. For 100% CDR efficiency, these values would be even lower at 12 years for TA-DIC, 5 years for pH, and 17 years for Ω_ar_ (Table S5). For the polar region and 80% CDR efficiency, this amounts to 21-, 18-, and 24-years prior, for TA-DIC, pH, and Ω_ar_, respectively. To have a mitigation time shift of more than a few years, the ΔTA must be higher than 10 μmol kg^–1^. Note that when mitigation times exceed 40 years, we cannot consider results to be accurate since the OceanSODA-ETHZ data is a 40-year record.

To calculate the mitigation time related to the biological response under equilibrated conditions, we applied the regression coefficients in Table 1 to the TA-DIC time series to estimate the calcification rates for the 1982–2022 period over both coastal and polar grid cells (80% CDR efficiency; Table S6; 100% CDR efficiency, Table S7). On average, different ΔTA additions (10, 50, and 100 μmol kg^–1^) led to a mitigation time of 4.5, 22, and 43 years, respectively, for the 80% scenario (Table S6), and 2, 11, and 23 years for the 100% scenario (Table S7). We show the calcification rate responses to TA addition for three selected species with diverse calcification patterns, i.e., two linear responders: the coral *Acropora yongei* and the gastropod *Strombus alatus*; and a threshold responder, the mollusk *Mercenaria mercenaria*, under both 80 and 100% CDR efficiency (Figures S9 and S10, respectively). The species *Acropora yongei* and *Strombus alatus* have different calcification rates and slopes, but the same mitigation times (46.2 years upon 100 μmol/kg TA addition) since they are both linear responders, whereas the threshold responder *Mercenaria mercenaria* has a mitigation time of 37.8 years upon the same TA addition.

#### 3.3.4 Regional TA Addition Required to Restore Preindustrial Conditions

To provide spatial context to the species-specific OAE required to restore preindustrial conditions, we used the OceanSODA-ETHZ gridded data set to estimate the same properties with global coverage. Considering both unequilibrated (CDR efficiency = 0%) and equilibrated (CDR efficiency = 80 and 100%) additions of NaOH and Na_2_CO_3_, up to double the amount of TA addition is required when considering equilibration with the atmosphere (Figure 8). For 0% CDR efficiency, up to approximately 75 μmol/kg of NaOH and 175 μmol/kg of Na_2_CO_3_ are needed to restore the system to preindustrial TA-DIC conditions. For equilibrated conditions, up to 150 μmol/kg NaOH and 250 μmol/kg Na_2_CO_3_ additions are in the high-latitude regions, the eastern equatorial Pacific, and the coastal upwelling regions such as the west coast of North and South America, where the buffer capacity is low, and the Revelle factor is high. Higher TA additions are generally required in the subtropical gyres, i.e., at over 200 and 300 μmol/kg for NaOH and Na_2_CO_3_, respectively, due to multiple factors, including higher restricted circulation, localized freshwater input, where the TA concentration and buffering capacity are higher (Figure 8 ^48^)

**Figure 8:**
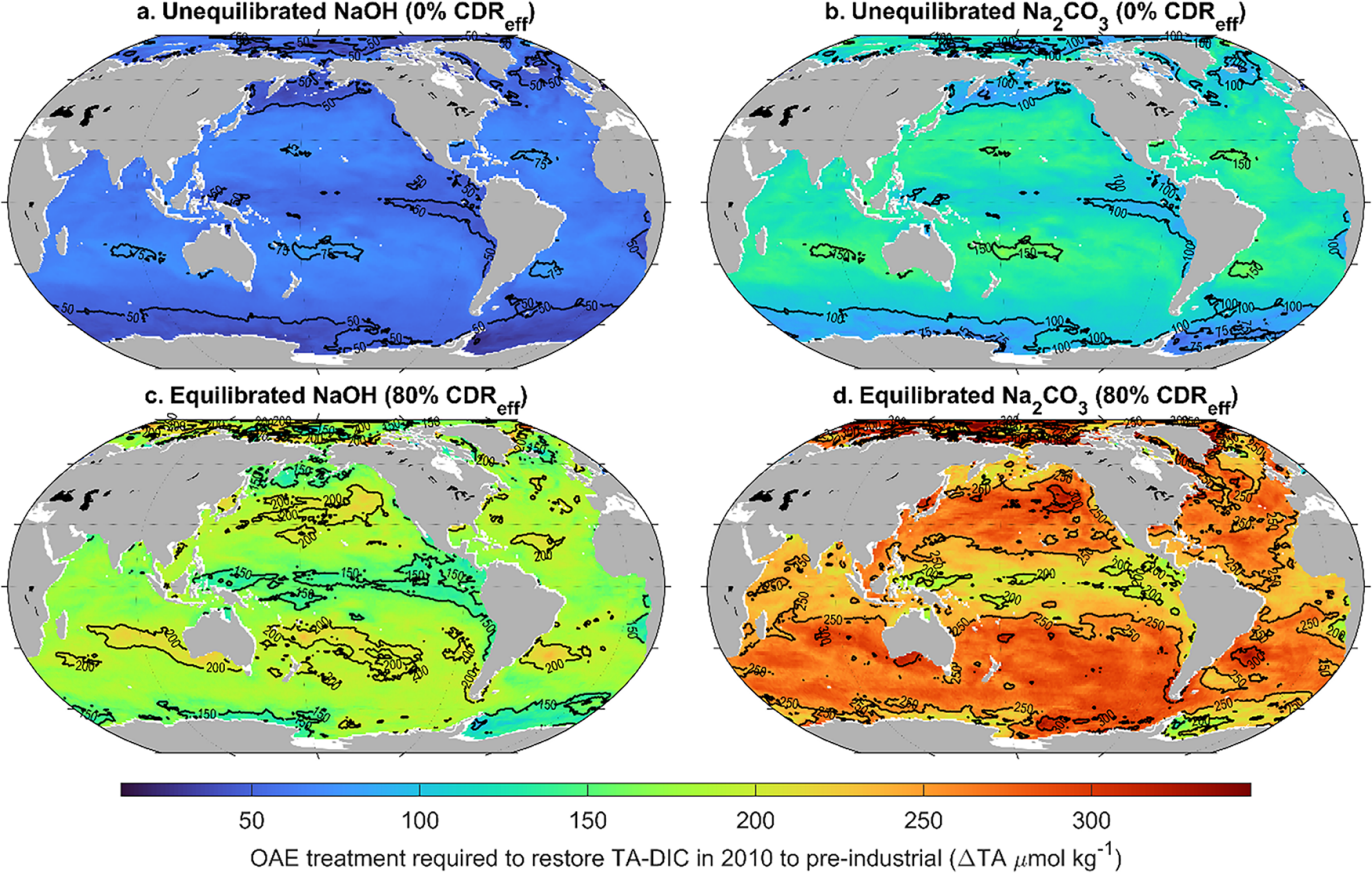
Global patterns in OAE treatments required to restore preindustrial TA-DIC levels. Global maps of the OAE treatments required to restore the TA-DIC of recent historical conditions (2010 represents the most recent historical conditions analyzed by Jiang et al.^48^) to preindustrial (1750) with (a) unequilibrated treatment using NaOH, (b) unequilibrated treatment using Na_2_CO_3_, (c) equilibrated treatment using NaOH assuming 80% CDR efficiency, and (d) equilibrated treatment using Na_2_CO_3_ assuming 80% CDR efficiency.

The unequilibrated and equilibrated values calculated per species’ baseline experimental conditions, in Tables 1 and S3 are generally higher than the results shown in Figure 8, especially for the corals *Solenastrea hyades*, *Siderastrea radians*, *Montastraea cavernosa*, and *Duncanopsammia axifuga*, and foraminifera *Marginopora vertebralis*, where these concentrations exceed 400 and 580 μmol/kg for equilibrated NaOH and Na_2_CO_3_, respectively.

## 4 Discussion

This study builds upon earlier conceptual frameworks evaluating the biological implications of OAE,^42^ and extends them by quantitatively linking species-specific calcification responses to dynamic TA-DIC perturbations under both equilibrated and nonequilibrated air–sea CO_2_ exchange scenarios. By coupling chemical and biological processes, this analysis provides a novel and scalable framework to assess how the efficiency of CO_2_ removal through the OAE interacts with its capacity to mitigate OA impacts on calcifying organisms.

The efficacy of the OAE as a strategy to mitigate changes in seawater–carbonate chemistry has been widely discussed, but the biological benefits of the OAE in this context have received less attention. We show that the biological benefits of OAE mitigation against OA are species-specific, and only practically relevant for OAE without additional CO_2_ uptake. As air-sea equilibration proceeds and variables controlling calcification return toward pretreatment levels (Figure 1), OA mitigation effects diminish. The more efficient the CO_2_ uptake (high CDR efficiency), the less effective OA mitigation of biological effects becomes. This creates an apparent contradiction: higher CDR efficiency enhances atmospheric CO_2_ drawdown but simultaneously reduces biological mitigation potential. Hence, it is essential to recognize that biological restoration will not necessarily align with climate mitigation. This duality, where the two processes act in opposing directions, poses challenges for implementing OAE in ways that realize biological cobenefits, with important implications for management.

### 4.1 Consideration of OAE for OA Mitigation Management

Strategies using the OAE to realistically and effectively restore and mitigate the biological effects of OA must account for the complex interplay between chemical conditions, CDR efficiency, and species-specific responses. Achieving high ecosystem restoration efficiency would require lower CDR efficiency and vice versa. This trade-off should guide the selection of habitats for OAE field trials in mitigating against OA, especially in areas severely impacted by OA, vulnerable habitats, such as coral and oyster reefs, nursery grounds for ecologically and economically important species, and sensitive aquaculture and fisheries sites. Importantly, the spatial scales of signal detection are likely limited to regional scales, which should be initiated as localized pilot projects that include modeling studies to assess the feasibility and ecological impacts before broader implementation in the field.

From the temporal perspective, preselecting habitats for OAE implementation should focus on periods where OA mitigation is critical, such as during sensitive early life stages or multiple-stressor events, although any comprehensive understanding on this is still missing. The underlying hypothesis is that reducing OA stress may free energetic resources that can be allocated to coping with other stressors. Although short mitigation times are not ideal, they may still offer benefits in extreme restoration or protection scenarios requiring critical OA relief. Future research for critical OA mitigation using the OAE should aim to identify strategies that optimize both outcomes whenever possible, including the selection of target regions, species, and deployment scales that align with defined restoration or mitigation priorities. However, subtle differences in the scope of the OAE mitigation remain species-specific, as discussed further below.

### 4.2 Variability in Species’ Calcification Response Determines Their OA Sensitivity

The variability in species’ calcification rate responses is a key factor in determining their sensitivity to OA. We provide a more detailed explanation of how the calcification strategy can determine calcification decline under OA and can be further interpreted in the context of OAE mitigation.

Species’ sensitivity is linked to their calcification strategy, which can be categorized as passive or active. The calcification of passively calcifying species is primarily governed by ambient seawater–carbonate chemistry owing to their limited ability to physiologically regulate the local chemical conditions at the site of calcification. These species typically lack mechanisms to elevate and keep pH, [CO_3_^2–^], or Ω_ar_ at the site of calcification stable, and therefore exhibit strong sensitivity to OA. Examples include certain foraminifera, coccolithophores,^59,60^ coralline algae^61^ and gastropods.^62^ In contrast, species capable of actively modulating their calcifying environment are better able to cope with OA because they regulate internal conditions via specialized compartments, enzymatic adjustments such as carbonic anhydrase activity,^63–65^ and other physiological mechanisms, allowing CaCO_3_ precipitation even under suboptimal external conditions. Examples include certain coccolithophores, corals, echinoderms, and some mollusks.^66–71^ Calcification response type (linear vs threshold) thus reflects the degree of biological control over the calcifying fluid.

In our study, the species with the largest decline in calcification rate were observed in passively calcifying species.^59,60^ The most sensitive species belong to the mollusk group, including gastropod and pteropod groups, with an average 23.6% drop in calcification rate since preindustrial times.^72,73^ Other mollusks showed a wide range of decline (0.4–44.4%), highlighting species-specific variability depending on calcification mechanisms, even within the same group.^62^ Species with >20% decline indicate passive calcification, while those with <5%, such as the Eastern oyster (*Crassostrea virginica*, 3.2% drop), suggest biologically mediated calcification or tighter control. These species can regulate the chemistry of their extrapallial fluid in the space between their mantle and shell to optimize conditions for shell formation, but it is likely that such compensation is short-lived and also not feasible during larval stages.^66^ Scleractinian corals (e.g., *Acropora* and *Pocillopora*) regulate the pH of their calcifying fluid (extracellular calcifying space) by actively transporting hydrogen ions (H^+^) out of the calcification site.^67,75^ However, the level of such regulation is likely species dependent, demonstrating variability of regulation capacities across the coral groups. This is further reflected in a range of calcification decline of 1.0 to 31.5%, where most of the species with stronger biological control show lower calcification decline up to 10%, compared to the species with less control capacity^61^ and thus higher calcification decline of 10 to 30% (*Siderastrea radians* and *Duncanopsammia axifuga*). Foraminifera show species-specific sensitivity: some species (*Globigerina bulloides*) regulate their calcifying microenvironment by selectively taking up bicarbonate and calcium ions from seawater, or adjust their intracellular pH to enhance carbonate availability,^74^ while the other species calcify passively.^72,73^ This is likely the reason that the foraminifera (*Marginopora vertebralis*) investigated in this study did not show any decline in calcification rate since preindustrial times. Still, we cannot exclude that the metabolic cost of such regulations cannot influence their resilience under a prolonged duration of unfavorable conditions.

There is a clear distinction between the linear and threshold responders in our study (see Figure 6a, where the threshold responders are indicated in bold). Linear responders demonstrate much greater response to OA since preindustrial times than threshold responders. However, we note that even within these two categories (linear vs threshold responders), different species vary in calcification response due to multiple parameters in the microenvironment, including cell-level pH control, enzyme activity (e.g., carbonic anhydrase), and ion transport capacity, which is then reflected in the variation of the magnitude of response. We found a greater variation in the magnitude of decline to OA in the group of linear responders, with some of them showing a relatively small decline in calcification to OA, which aligns with better physiological control compared to the other linear responders with massive calcification decline and corresponding poor control. On the other hand, for the threshold responders, the pattern of decline is more reliable, with the species consistently showing small calcification decline and less variation between the species. This is a generalizable pattern that holds true across the experimental carbonate chemistry ranges examined, where the calcification response to the OAE relates to its calcification mechanism. Depending on the spacing of treatments, light level, exposure duration, and species life stage, the resulting regressions may be different. This means response type captures empirical behavior across experimental carbonate chemistry gradients, not exclusively an innate biomineralization strategy.

Additional outcome of the study reveals no observed difference in calcification to OA between photosynthetic and nonphotosynthetic calcifying taxa. Although photosynthetic calcifying organisms can increase pH within their diffusive boundary layer, these effects are local, transient, and variable, with the magnitude of this increase depending strongly on light intensity, flow, morphology, and metabolic rates. As a result, its influence on calcification is dependent on the conditions in the internal environment of the organisms and is not a species-intrinsic trait. Rather, the actual calcification depends on whether the species can control the pH, DIC, and ion gradients within the calcifying space. As such, photosynthesis does not equate to strong biological control of the calcification site, and the determinant of response type is the calcifying physiology. Many photosynthetic calcifiers (e.g., some coralline algae) remain passive internal calcifiers, while some nonphotosynthetic species (e.g., corals, some mollusks) exert strong active regulation through ion transport. As such, threshold vs linear categorization reflects response to carbonate chemistry constraints and does not partition distinctly between photosynthetic vs nonphotosynthetic taxa. An additional reason for this could also be that the regression models in this study are based on experimental carbonate chemistry treatments, where light as an experimental parameter is mostly not included as a variable. Such treatments reflect physiological sensitivity to TA-DIC while minimizing light-driven photosynthetic pH effects. These two reasons mostly explain why photosynthetic vs nonphotosynthetic status did not map onto calcification response type.

### 4.3 Scenarios to Increase the Calcification Rate upon TA Addition

The OA mitigation potentials vary substantially among different calcifying groups and species. Since passive calcifying species have been most affected by OA, they are also the group that will benefit the most from OAE due to their reliance on the passive uptake of carbonate ions (increased TA-DIC). On the other hand, linear responders with biologically mediated calcification and smaller loss in calcification rate since preindustrial times are likely to benefit less. The third group of responders are the species with threshold-type calcification responses (Table 1) that were minimally affected by OA since preindustrial times. Across all of the threshold responders, the decline in calcification was less than 10%, suggesting that TA addition may not play a significant direct role in mitigation for those species. However, these organisms may still benefit indirectly as less energy would be required to maintain optimal calcification, an important aspect that warrants further experimental investigation.

While many species show similar responses in calcification to both OA and OAE, some, such as *Duncanopsammia axifuga* and *Montastrea cavernosa*, exhibit lower responsiveness to OAE than expected, shown by high TA concentrations of over 800 μmol/kg NaOH and almost 1200 μmol/kg Na_2_CO_3_ addition required to restore preindustrial conditions, and the lower calcification increase upon enhancement of TA by a fixed concentration, compared to the rate of OA calcification rate decline. This is at least partially due to the baseline natural environments (assumed similar to experimental control conditions) that have a high buffer capacity with a high TA (2371 μmol kg^–1^ and a low DIC of 1885 μmol kg^–1^).

Our findings show that very high concentrations of TA are needed to reach preindustrial conditions, indicating that the return to preindustrial conditions and calcification is highly unlikely. Addition of TA in the more realistic ranges (less than 50 μmol kg^–1^) may not be able to restore preindustrial conditions, but can still mitigate the negative biological effects of OA to some extent in localized areas near the injection site. When considering the mitigation time of OAE to restore chemical conditions, an addition of 50 μmol kg^–1^ of NaOH without re-equilibration with the atmosphere, the OA mitigation effect corresponds to about 20 years in the coastal region under current OA rates (see Table S5), and lower dosages recover less than a year, indicating very low mitigation potential.

Our results apply to both near-field and far-field impacts of OAE interventions.^76^ Our results prior to equilibration with the atmosphere are directly relevant for the near-field conditions, where higher TA may create biological exposure with some potential benefits for OA mitigation. However, there is a risk for unforeseen negative ecological impacts, which still remain a concern in the near-field under much higher, unequilibrated OAE additions.^42^ On the other hand, our postequilibration results are relevant for the far-field OAE implementation. In the far-field, biological mitigation effects are smaller but negative side effects are also reduced.

#### 4.3.1 Comparison of Biological and Chemical OA Mitigation

The biological interpretation of the mitigation of OAE is closely linked to chemical OA-mitigation dynamics, particularly over large and longer-term scales, and less so over short or local spatial scales. Our results suggest that the temporal and spatial extent of OAE as a mitigation capacity for OA is variable and species dependent. Recent studies report OAE levels similar to our findings, indicating that TA enhancements of at least ∼10 μmol kg^–1^ are needed to produce detectable mitigation. A study analyzing the continuous release of TA over one year along the north–south extent of the Great Barrier Reef showed that the projected Ω_ar_ would increase by about 0.1 to 0.15 with ΔTA 0–30 μmol kg^–1^ corresponding to a 2.85 × 10^8^ mol TA addition yr^–1^.^25^ Similarly, a point-source OAE study in Unimak Pass, Alaska, found pH increases of <0.02 near the mitigation site with ΔTA 0–3 μmol kg^–1^ upon 1.67 × 10^10^ mol TA addition yr^–1^.^26^

### 4.4 Uncertainties Related to the Experimental Conditions

The carbonate-chemistry conditions used in the experimental treatments, particularly the baseline control conditions, play a central role in evaluating the uncertainty of the predicted results. Species response variability determines the overall signal-to-noise ratio. Notably, experiments using a narrow range of carbonate chemistry conditions (e.g., only two treatment levels), tend to introduce substantial uncertainty when extrapolating to higher unequilibrated TA additions (>200 μmol kg^–1^), which fall outside the tested range. This uncertainty is less relevant for unequilibrated scenarios, where the TA additions are typically smaller.

In addition, control of experimental temperature to determine both current and preindustrial conditions could also impact uncertainty, as wider temperature ranges can potentially introduce additional variability in studies where calcification is temperature-sensitive.^81^ While the temperature range (Δ*T*, calculated as the difference between maximum and minimum temperatures) was especially large for six species (*Siderastrea radians*, *Solenastrea hyades*, *Crassostrea virginica*, *Limacina helicina*, *Concholepas concholepas*, and *Mytilus edulis*), ranging from 3.7–12 °C, for all the other species Δ*T* was less than 0.6 °C. Thus, temperature effects likely did not substantially influence the biological results, consistent with several studies reporting minimal temperature impacts.^82,83^ Additional, less quantifiable uncertainty arises because we did not account for long-term changes in temperature or TA since the preindustrial period, assumed a uniform ηmax across coastal regions, and neglected atmospheric CO_2_ alteration by OAE under equilibrated conditions.

We also assume that calcification responses to TA-DIC changes are locally reversible and symmetric; i.e., sensitivity to increasing TA-DIC mirrors the sensitivity observed during decreases. We acknowledge that this simplification overlooks potentially more complex hysteresis-like dynamics in which recovery may follow a different physiological pathway than stress exposure. However, this cannot be resolved from the available OA data sets and assuming reversibility without hysteresis is a necessary and reasonable simplification for quantifying the immediate mechanistic sensitivity of calcification to the OAE. Our analysis remains within a relatively small TA-DIC window where responses are well approximated by the fitted regressions and do not extend far into domains where nonlinear hysteresis would be expected. Given the relatively short biological time scales of calcification relative to the slow trends in surface ocean TA-DIC, it is reasonable to treat responses as near-instantaneous with respect to changes in external carbonate chemistry.

### 4.5 Implications

Our findings provide new insights into the potential and limitations of the use of the OAE as a strategy to mitigate OA, both chemically and biologically. We find that OAE can partially mitigate OA’s biological effects, though this requires high TA concentrations, especially when considering atmospheric equilibration. Effectively mitigating biological impacts requires an OAE at low CDR efficiency, a trade-off that compromises the intervention’s original goal and cannot be controlled once TA is released. The ecological impacts of OAE must be carefully evaluated, as it may alter marine biogeochemical cycles and affect species in various ways.^15,86^ Future experimental and modeling studies should incorporate conditions that improve the understanding of calcification processes, including light- and temperature-dependent sensitivities, longer-term adaptation responses, and the influence of temporal and spatial variability and mixing. Many processes affecting TA and the carbonate system, such as calcification or dissolution feedbacks, remain understudied due to limited data and parametrization. This is important because calcification counteracts OAE’s intended effect of the OAE, reducing efficiency. However, increased calcification in pelagic calcifiers could also raise sinking velocities of particulate organic carbon, enhancing CO_2_ uptake,^84,85^ though it is currently uncertain if OAE can trigger positive or negative feedback loops. Addressing these gaps will require interdisciplinary efforts that integrate experiments, modeling, and field studies to better understand and optimize the role of the OAE in mitigating OA. Ultimately, integrating the OAE with a broader suite of carbon strategies could enhance its overall effectiveness as part of a multipronged approach to mitigating climate change impacts.

## Supplementary Material



## Data Availability

The data set compiled in this study is a subset that has been previously used for the evaluation of OAE effects in biogeosciences (Bednaršek et al., 2025). This data set, as well as all other data and code related to this study, is available in the Github repository at https://github.com/hannavdmortel/OAE_mitigation_of_OA (last access: 16 May 2025) and is archived on Zenodo at 10.5281/zenodo.15394079 (van de Mortel, 2025). PyCO2SYS v1.8.0 (Humphreys et al., 2022) was used to solve for the carbonate system, with software available at 10.5281/zenodo.3744275 (Humphreys et al., 2024). PyOAE (https://github.com/gjpelletier/PyOAE) is a Python package developed during this study that is equivalent to the Matlab scripts that were used to solve for the OAE treatment needed to restore OA indicators to preindustrial conditions and the response of ηmax to OAE.

## Notes

Additional experimental details and computational methods; sensitivity analysis of marine calcifiers to carbonate chemistry changes; OA data compilation; preindustrial seawater–carbonate state calculations; equilibrium computations for OAE scenarios with and without atmospheric CO_2_ uptake; TA addition requirements; prediction intervals; estimates of OAE additions needed to restore current conditions to preindustrial levels; figures illustrating carbonate chemistry responses to NaOH and Na_2_CO_3_ addition, species functional group distributions, global regressions, ηmax sensitivity and regional maps, calcification rate responses under varying TA additions and CDR efficiencies; tables summarizing ηmax values, species collection locations, regression metrics, Na_2_CO_3_ addition requirements, and time mitigation analyses for TA-DIC, pH, Ω_ar_, and calcification rates (PDF)

## ○

Retired.
