# Supplementary material for: Substantial Limitations of Ocean Alkalinity Enhancement in Mitigating the Negative Impacts of Ocean Acidification on Marine Calcifiers
Source: Environ Sci Technol. 2025 Dec 28;60(1):622–41. doi: 10.1021/acs.est.5c09298 (PMC12810250; doi:10.1021/acs.est.5c09298)
Supplement: Supplementary file 1 [file es5c09298_si_001.pdf]

Supporting Information

Substantial limitations of ocean alkalinity enhancement in mitigating the negative impacts of ocean acidification on marine calcifiers

Hanna van de Mortel[1,2#], Nina Bednaršek[3*#], Greg Pelletier[4☆], Richard A. Feely[5], Jens D. Müller[6,7], Nicolas Gruber[6]

[1]*HvdMortel Consulting, Utrecht, 3515GS, Netherlands;*

[2] *Institute for Marine and Antarctic Studies, University of Tasmania, Hobart, TAS 7004, Australia*

[3]*Jožef Stefan Institute, Environmental Department, Ljubljana, 1000, Slovenia*

[4]*Washington Department of Ecology, 300 Desmond Dr SE, Lacey, WA 98503, USA;*

[5]*Pacific Marine Environmental Laboratory, NOAA, 7600 Sand Point Way NE, Seattle, WA 98037, USA;*

[6]*Environmental Physics, Institute of Biogeochemistry and Pollutant Dynamics, ETH Zurich, Zurich, 8092, Switzerland;*

[7]*Carbon to Sea Initiative, 1828 L St NW, Suite 300-C, Washington D.C., 20036, USA*

*Email: nina.bednarsek@ijs.si

[#]Hanna van de Mortel and Nina Bednaršek contributed equally to this work.

[☆]Retired

This PDF file includes:

Supplementary Methods S1-S7

Figs. S1 to S11

Tables S1 to S7

Supplementary Methods

S.1 Sensitivity of marine calcifiers to changes in carbonate chemistry

To evaluate the potential of OAE to restore calcification rates, we applied species-specific regression models derived from experimental OA calcification data (*42*), whereby we used TA-DIC as the independent variable. TA-DIC, also referred to as 'Alk*' in other studies (*43*), explains over 99% of the variability in [CO_3^{2-}] and aragonite saturation states (Ω_{ar}), which are directly related to calcification (Figure S2). It is also closely related to the TA:DIC variable, which has been applied in a similar study by Bednaršek et al. (*42*). We selected the TA-DIC variable as it allows for the most direct assessment of changes in the marine carbonate system upon changes in DIC, TA or both. Two types of regression models to the experimental data were used for each species to group them into two response types: (i) linear, where calcification rates increase proportionally with TA-DIC, and (ii) threshold (exponential regression), where calcification rates remain constant before dropping when a critical TA-DIC value is reached. Note that this study only examines species with a positive correlation between calcification rate and TA-DIC. Figure 2 shows both response types, with the experimental data and respective regression models shown alongside their prediction intervals. We applied an ordinary least squares (OLS) regression model (*44*), selecting the model with the lowest p-value, considering only models with $p < 0.05$ as significant.

S.2 OA data compilation

The experimental data used in this study consist of existing studies on marine species calcification response previously published in Bednaršek et al. (*42*). The experimental data used in this study consist of existing studies on marine species calcification response that aligned calcification rate

data along with carbonate chemistry, and were previously compiled by Bednaršek et al. (*42*). Their compiled dataset covered a wide range of calcifying organisms across various functional groups and 84 species. We examined 27 species that inhibit surface waters and have shown positive responses to OAE, with experimental control concentrations of TA and DIC deemed representative of their natural environmental conditions. Of these 27 species, 20 are linear and 7 are threshold responders. They cover a wide range of functional groups: calcifying algae, corals, crustaceans, echinoderms, foraminifera, gastropods, pteropods and (other) mollusks (Figure S2). Note that the group of gastropods refers to all gastropods that are not pteropods. Most of the functional groups investigated here use carbonate ions for calcification, making it directly comparable to the TA-DIC (which approximates carbonate ion concentration; Figure S3), with only a few exceptions for the phytoplankton autotrophs that also use bicarbonate ions. A variety of calcification rate units were used across different studies, and these were standardized where possible. This standardization is thoroughly explained by Bednaršek et al. (*42*). For single-cell organisms, growth rates and PIC production rates were used as indicators of calcification rate. For some species, direct calcification rates were not reported in the literature and only relevant parameters related to calcification (shell length, density, thickness) over time were available. While this data was also collected by Bednaršek et al. (*42*), it was not used in this study. Where there were multiple studies available for the calcification rate of one species using the same rate units, the data were combined. Data were analyzed on a species level, wherever rate units were the same. TA-DIC on the x-axis strongly correlates to [CO_3^{2-}] (Figure S3a).

S.3 Computation of the pre-industrial state of seawater carbonate chemistry

We define a current conditions baseline based on species-specific experimental control TA, DIC, temperature and salinity. We assume experimental control conditions to be representative of

natural conditions in each species' typical habitat. From TA and DIC, we derived the current mean pCO_2 values of the species' natural conditions. To estimate pre-industrial conditions, we assumed pCO_2 was 142 ppm lower than today, based on the difference between the global current atmospheric pCO_2 of 420 ppm (*1*) and pre-industrial pCO_2 of 278 ppm (*45*), reflecting that – in the long-term – the growth in seawater pCO_2 tends to closely follow the growth in the atmospheric pCO_2, while maintaining the regional air-sea disequilibrium. This methodology is visualized in Step 1 of Figure 1b, where the origin of the three arrows represents the current conditions. We assume TA has not changed since the pre-industrial era, which is supported by only minor observed changes in TA over the past 40 years (*46*, *47*), and by model studies indicating the stability of TA over the industrial era (*48*). Since we assume TA has remained constant since the pre-industrial era, the change in TA-DIC is equal to the change in DIC. Likewise, the experimental control temperature and salinity was used to compute the pre-industrial conditions, meaning their long-term changes are not considered. Carbonate system calculations were done in CO2SYS (*19*), using $[Si(OH)_4]$ = 50 μmol kg^{-1}, $[PO_4^{3-}]$ = 0.5 μmol kg^{-1} and the stoichiometric dissociation constants for carbonic acid from Lueker et al. (*20*), for sulfuric acid by Dickson et al. (*21*) and for total boron from Lee et al. (*22*).

S.4 Equilibrium computation of the marine carbonate system for OAE with and without CO_2 uptake from the atmosphere

The theoretical maximum OAE efficiency (ηmax) is defined by Yankovsky et al. (*49*) as the maximum increase in moles of DIC that could occur through mCDR for a given increase in moles of TA through OAE. With ηmax we can also compute the CDR efficiency, which describes the effectiveness of actual atmospheric CO_2 removal relative to ηmax (*51*). Unlike ηmax, which can be estimated from static carbonate chemistry reconstructions, the quantification of CDR efficiency

requires dynamic modeling of air-sea gas exchange processes to capture the gradual equilibration of CO_2 between the ocean and atmosphere. Hence, we adopted CDR efficiency estimates from the literature for our offline computations (see below). Full methodological details, including equations and computational procedures, are provided in Supplementary Text S1. Recent studies indicate CDR efficiency to be location and time dependent, with lower values ranging between 0.2 and 0.85 over the first few years of continuous OAE loading, and between 0.65 and >0.95 afterwards (*26*, *51*, *52*) or with the estimates on the global scale of 0.71 - 0.84 (*53*). As such, we have chosen CDR efficiency to be 80% for our analysis, which aligns well with the estimates by Yamamoto et al. (*54*).

S.5 TA additions required to restore pre-industrial conditions

For our first approach, we computed the required amount of TA added to restore pre-industrial TA-DIC (Step 2 or 3 of Figure 1b). While this actual concentration of TA required is shown in Tables 1 and S4, on our figures we have added four increments of TA (ΔTA; in steps of 50 μmol kg^{-1}) from the current conditions baseline, assuming unequilibrated conditions (see Figure 2). Note that the amount of TA required to restore pre-industrial conditions is not biologically controlled– it is solely dependent on the experimental TA, salinity, and temperature. Some experiments were carried out over a range of temperatures and salinities, introducing greater uncertainty (ranges are shown in Table S3). While this actual concentration of TA required is shown in Tables 1 and S4, on our figures we have added four increments of TA (ΔTA; in steps of 50 μmol kg^{-1}) from the current conditions baseline, assuming unequilibrated conditions (see Figure 2). This is solely to visualize this conceptual addition of TA and give a visual indication of the magnitude of TA addition to restore pre-industrial conditions.

S.6 Prediction intervals

Prediction intervals were calculated for each regression model based on experimental data, best-fit model parameters, prediction points, and a significance level of 0.1 (yielding 90% intervals). Prediction intervals account for model uncertainty and biological variability in the OA experiments and are especially important when estimating values beyond the observed data range. This interval was computed using a two-tailed t-distribution to derive critical t-values, which define the upper and lower bounds around the predicted values. These bounds reflect the expected range of the mean or individual responses at each prediction point and are summarized in Table 1 for current and pre-industrial conditions. To support interpretation of model reliability, residual sum of squares, residual mean square error, and standard error of the estimate are provided. These metrics quantify the unexplained variance and help evaluate the accuracy and predictive strength of the regression fit.

S.7: Estimates of the amount of OAE addition needed to restore the current conditions (year 2010) to pre-industrial conditions (year 1750)

The difference between TA and DIC, also known as Alk* *(37)*, can be used to interpret the response of calcification to OA and OAE *(36)*. In this manuscript we use TA-DIC as the independent variable in regression analysis of calcification rates for various species as the dependent variable.

Ocean acidification has caused a decrease in TA-DIC since pre-industrial conditions *(37)*. One of the questions we attempt to address in our manuscript is the quantity of OAE required to restore the recent historical TA-DIC to pre-industrial conditions.

The following general equations describe the condition when the quantity Alk* = TA-DIC after OAE treatment at any given time t (e.g. t = year 2010) is equal to the pre-industrial condition (e.g. year 1750), for either un-equilibrated or equilibrated conditions, and for OAE treatment with either NaOH or Na_2CO_3:

$$(TA_t + \Delta TA_{trt}) - (DIC_t + \Delta DIC_{trt} + \Delta DIC_{cdr}) = TA_{PI} - DIC_{PI} \quad (1)$$

$$Alk^*_t + \Delta TA_{trt} - \Delta DIC_{trt} - \Delta DIC_{cdr} = Alk^*_{PI} \quad (2)$$

$$Alk^*_t - Alk^*_{PI} + \Delta TA_{trt} - \Delta DIC_{trt} - \Delta DIC_{cdr} = 0 \quad (3)$$

Note that ΔDIC_{trt} and ΔDIC_{cdr} can be expressed in terms of ΔTA_{trt} as follows:

$$\Delta DIC_{trt} = 0 \text{ when NaOH is used for the OAE treatment } (\mu mol\ kg^{-1}) \quad (4)$$

$$\Delta DIC_{trt} = 0.5\ \Delta TA_{trt} \text{ when } Na_2CO_3 \text{ is used for the OAE treatment } (\mu mol\ kg^{-1}) \quad (5)$$

$$\Delta DIC_{cdr} = 0 \text{ for un-equilibrated conditions} \quad (6)$$

$\Delta DIC_{cdr} = CDR_{eff}\ \eta max\ \Delta TA_{trt}$ (μmol kg^{-1}) for equilibrated conditions (7)

Where

TA_t = TA at time t before treatment (μmol kg^{-1})

DIC_t = DIC at time t before treatment (μmol kg^{-1})

TA_{PI} = pre-industrial TA (μmol kg^{-1})

DIC_{PI} = pre-industrial DIC (μmol kg^{-1})

$Alk^*_t = TA_t - DIC_t$ at time t before OAE treatment (μmol kg^{-1}) (8)

$Alk^*_{PI} = TA_{PI} - DIC_{PI}$ at pre-industrial conditions (μmol kg^{-1}) (9)

ΔTA_{trt} = directly added TA due to OAE treatment (μmol kg^{-1})

ΔDIC_{trt} = directly added DIC due to OAE treatment (μmol kg^{-1})

ΔDIC_{cdr} = indirectly added DIC due to CDR (μmol kg^{-1})

CDR_{eff} = CDR efficiency (dimensionless, e.g. CDR_{eff}=0.8 if CDR efficiency is 80%)

$CDR_{eff} = \eta(t) / \eta max$ (10)

Where $\eta(t)$ = realized DIC increase due to CDR after OAE (dimensionless), and ηmax = hypothetical maximum DIC increase at equilibrium due to CDR after OAE (dimensionless), calculated as follows with CO2SYS using control conditions of pCO2 combined with treatment conditions of TA to estimate the DIC at equilibrium (DIC_{eq}):

$\eta max = (DIC_{eq} - DIC_t) / \Delta TA_{trt}$ (11)

Figure S4a shows the ηmax calculated using CO2SYS with control conditions of pCO_2 before treatment at time t, combined with treatment conditions of $TA = TA_t + \Delta TA_{trt}$, assuming $\Delta DIC_{trt}=0$, and using ΔTA_{trt}= 1 μmol kg^{-1} , using data from Jiang et al. *(48)* in the year 2010.

Figure S4b shows the difference in ηmax calculated assuming ΔTA_{trt}= 100 μmol kg^{-1} compared with ηmax calculated assuming ΔTA_{trt}= 100 μmol kg^{-1}. Figure S4b shows that ηmax is not very sensitive to the magnitude of ΔTA_{trt} in the range of 1-100 μmol kg^{-1}, with an average relative difference (Δηmax) of around <0.2%. This finding is consistent with results reported by Yankovsky et al. *(51)*, who reported a global average variation in ηmax of <0.1% using ΔTA_{trt} in the range of 1-100 μmol L^{-1}.

If NaOH is used for OAE treatment, then all of the increase in DIC from OAE is attributable to CDR. If Na_2CO_3 is used for the OAE treatment, then equation 5 describes ΔDIC_{trt}, which is the part of the increase in DIC that is due to direct chemical addition. Therefore, the hypothetical maximum increase in DIC attributable to CDR is estimated as follows when Na_2CO_3 is used for OAE treatment:

$$\eta max_{Na2CO3} = (DIC_{eq} - DIC_t - \Delta DIC_{trt}) / \Delta TA_{trt} \quad (12)$$

Substituting equation 5 into equation 12 results in the following:

$$\eta max_{Na2CO3} = (DIC_{eq} - DIC_t - 0.5\ \Delta TA_{trt}) / \Delta TA_{trt} \quad (13)$$

equation 13 simplifies to the following:

$$\eta max_{Na2CO3} = (DIC_{eq} - DIC_t) / \Delta TA_{trt} - 0.5 \quad (14)$$

$$\eta max_{Na2CO3} = \eta max - 0.5 \quad (15)$$

OAE treatment using NaOH under un-equilibrated conditions

$\Delta DIC_{trt} = 0$, and $\Delta DIC_{cdr} = 0$, therefore substituting into equation 2 simplifies to the following:

$$Alk^*_t + \Delta TA_{trt} = Alk^*_{PI} \quad (16)$$

$$\mathbf{\Delta TA_{trt} = Alk^*_{PI} - Alk^*_t} \quad (17)$$

<u>OAE treatment using Na_2CO_3 under un-equilibrated conditions</u>

$\Delta DIC_{trt} = 0.5\ \Delta TA_{trt}$, and $\Delta DIC_{cdr} = 0$, therefore substituting into equation 2 simplifies to the following:

$$Alk^*_t + \Delta TA_{trt} - 0.5\ \Delta TA_{trt} = Alk^*_{PI} \quad (18)$$

$$\mathbf{\Delta TA_{trt} = 2\ (Alk^*_{PI} - Alk^*_t)} \quad (19)$$

Figure 7a-b show the results of equation 17 (for NaOH) or equation 19 (for Na_2CO_3) to solve for the OAE treatments needed to restore Alk* in 2010 to pre-industrial, assuming un-equilibrated conditions.

<u>OAE treatment using NaOH under equilibrated conditions</u>

$\Delta DIC_{trt} = 0$, and $\Delta DIC_{cdr} = CDR_{eff}\ \eta max\ \Delta TA_{trt}$ ($\mu mol\ kg^{-1}$), therefore substituting into equation 2 simplifies to the following:

$$\Delta TA_{trt} - CDR_{eff}\ \eta max\ \Delta TA_{trt} = Alk^*_{PI} - Alk^*_t \quad (20)$$

$$\Delta TA_{trt}\ (1 - CDR_{eff}\ \eta max) = Alk^*_{PI} - Alk^*_t \quad (21)$$

$$\mathbf{\Delta TA_{trt} = (Alk^*_{PI} - Alk^*_t) / (1 - CDR_{eff}\ \eta max)} \quad (22)$$

Figure 7c shows the result of application of equation 11 and 22 iteratively using CO2SYS to solve for the corresponding values of ηmax and ΔTA_{trt} in each iteration, with initial ηmax from Figure S4 and initial ΔTA_{trt} using equation 11 in the first iteration. Each subsequent iteration uses the

ηmax and ΔTA_{trt} values from the previous iteration. The iterative solution approaches zero for the right hand side of equation 3. Convergence of the solution with RMSE <1e-6 µmol kg^{-1} across all grid cells, comparing treated Alk^*_{2010} with Alk^*_{PI}, occurred after five iterations, which was considered sufficient for reasonable precision.

OAE treatment using Na_2CO_3 under un-equilibrated conditions

$\Delta DIC_{trt} = 0.5\ \Delta TA_{trt}$, and $\Delta DIC_{cdr} = CDR_{eff}\ \eta max_{Na2CO3}\ \Delta TA_{trt}$ (µmol kg^{-1}), therefore substituting into equation 2 simplifies to the following:

$$Alk^*_t + \Delta TA_{trt} - \Delta DIC_{trt} - \Delta DIC_{cdr} = Alk^*_{PI} \quad (23)$$

$$Alk^*_t + \Delta TA_{trt} - 0.5\ \Delta TA_{trt} - CDR_{eff}\ \eta max_{Na2CO3}\ \Delta TA_{trt} = Alk^*_{PI} \quad (24)$$

$$0.5\ \Delta TA_{trt} - CDR_{eff}\ \eta max_{Na2CO3}\ \Delta TA_{trt} = Alk^*_{PI} - Alk^*_t \quad (25)$$

$$\Delta TA_{trt}\ (0.5 - CDR_{eff}\ \eta max_{Na2CO3}\ ) = Alk^*_{PI} - Alk^*_t \quad (26)$$

$$\Delta TA_{trt} = (Alk^*_{PI} - Alk^*_t)\ /\ (0.5 - CDR_{eff}\ \eta max_{Na2CO3}\ ) \quad (27)$$

$$\mathbf{\Delta TA_{trt} = (Alk^*_{PI} - Alk^*_t)\ /\ (0.5 - CDR_{eff}\ (\eta max - 0.5))} \quad (28)$$

Figure 7d shows the result of application of equation 15 and 28 iteratively using CO2SYS to solve for the corresponding values of ηmax and ΔTA_{trt} in each iteration. Convergence of the solution with RPD < 0.01% occurred after four iterations.

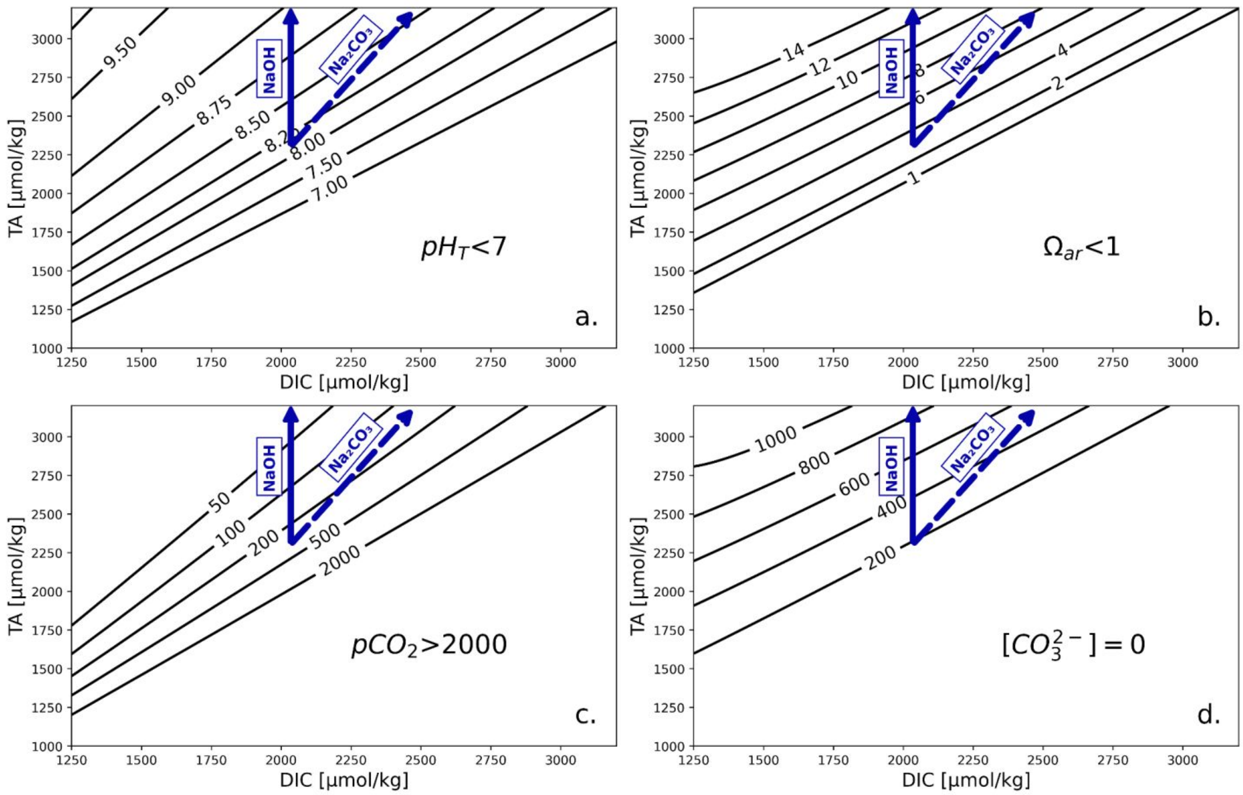

Fig. S1: Carbonate chemistry response to NaOH and Na_2CO_3 addition. The effect of changes in TA and DIC on the properties of seawater (S= 34.68, T=16°C, $[Si(OH)_4]$ = 50 µmol kg^{-1}, $[PO_4^{3-}]$ = 0.5 µmol kg^{-1}), adapted from Schulz et al. *(100)*. Subfigures show pH_T, Ω_{ar}, $[CO_3^{2-}]$ and pCO_2 concentrations. Calculations were carried out with PyCO2SYS, the Python version of CO2SYS *(42)*, using the stoichiometric dissociation constants for carbonic acid from Lueker et al. *(43)*, for sulfuric acid by Dickson et al. *(44)* and for total boron from Lee et al. *(45)*. The solid white line indicates the effect of adding NaOH and the dashed white line indicates the effect of adding Na_2CO_3, starting at initial conditions of TA = 2303 µmol kg^{-1} and DIC = 2034 µmol kg^{-1}. This line can be translated so that its initial position moves elsewhere to visualize different initial conditions. Note that at TA < 1000 µmol kg^{-1} and DIC < 500 µmol kg^{-1} the isolines are no longer straight when considering Ω_{ar}, however, such conditions are rare in the ocean and not widely applicable.

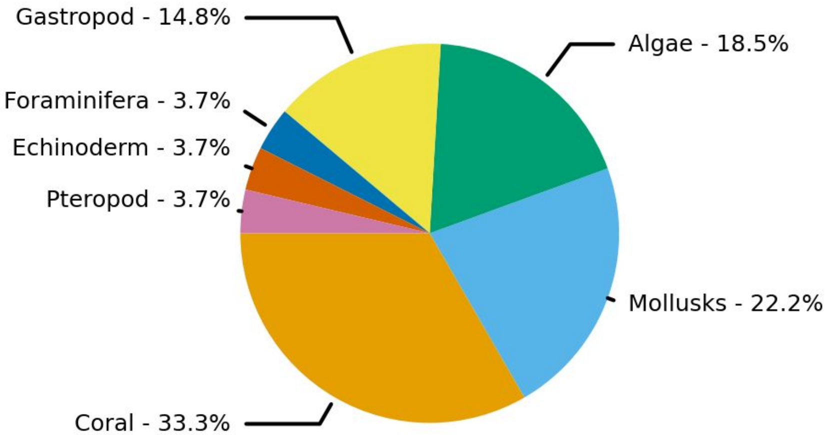

Fig. S2: Functional group distribution among studied marine calcifiers. Pie chart representing the relative distribution of 27 marine calcifying species across seven functional groups.

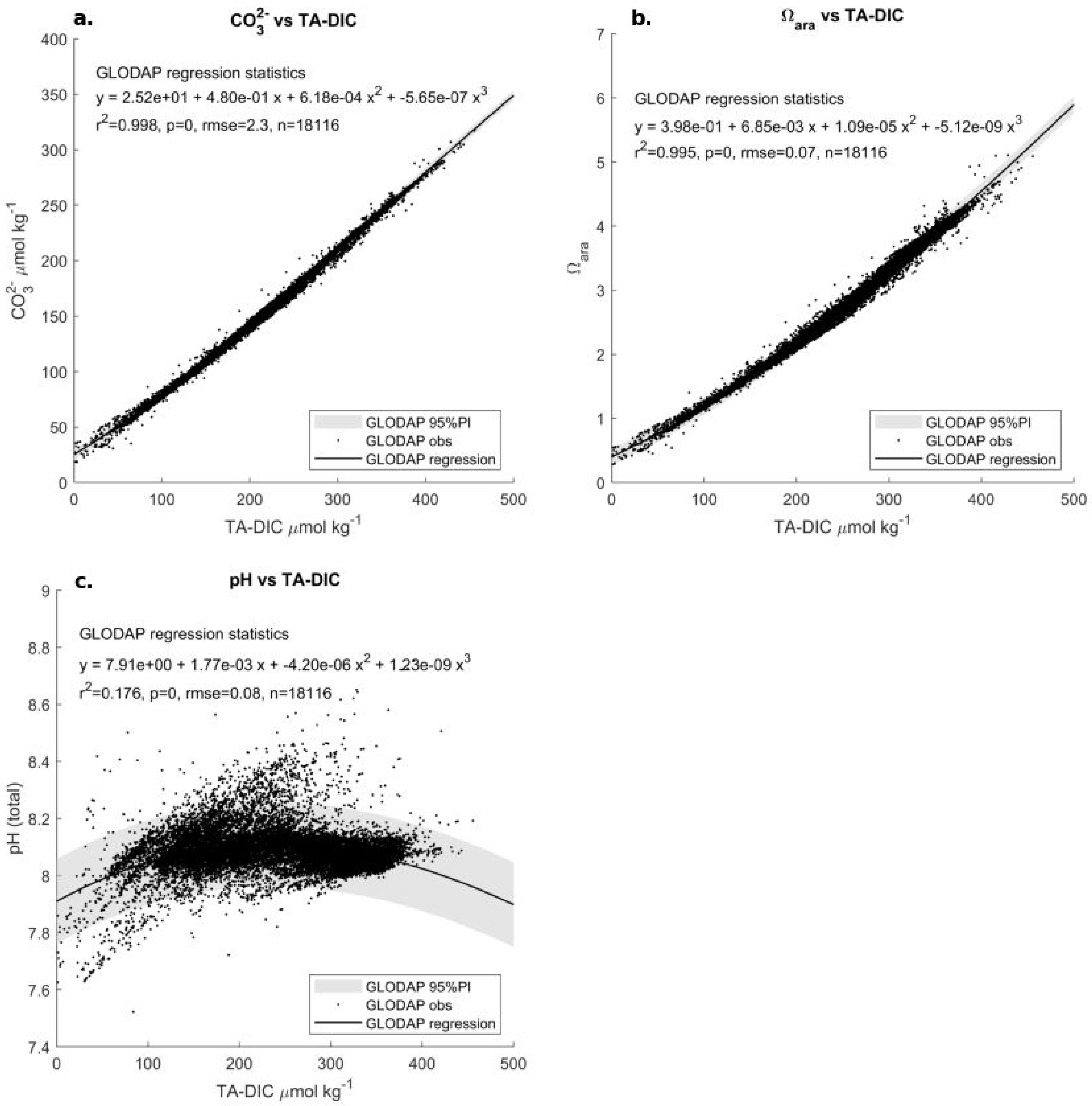

Fig. S3: CO_3^{2-}, Ω_{ar} and pH against TA-DIC. Regressions of observed CO_3^{2-}, Ω_{ar} and pH against TA-DIC globally for 0-10m depth in GLODAPv2.2023.

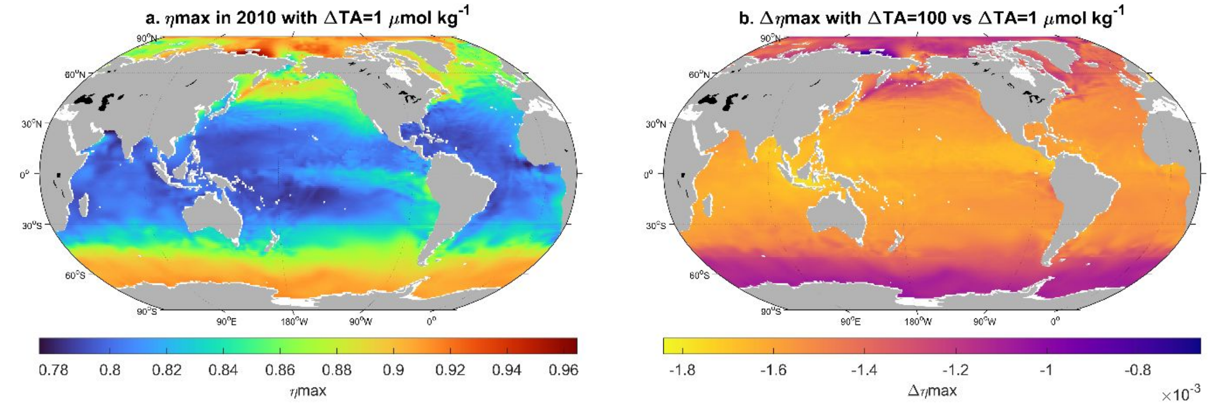

Fig. S4: Sensitivity of ηmax to ΔTA. Global maps of a) ηmax is calculated using CO2SYS with control conditions of pCO_2 before treatment at time t, combined with treatment conditions of TA = $TA_t+\Delta TA_{trt}$, assuming $\Delta DIC_{trt}=0$, and using ΔTA_{trt}= 1 μmol kg^{-1}, using data from Jiang et al. *(48)* in the year 2010, and b) the difference in ηmax comparing the assumption of ΔTA =100 μmol kg^{-1} vs ΔTA=1 μmol kg^{-1}.

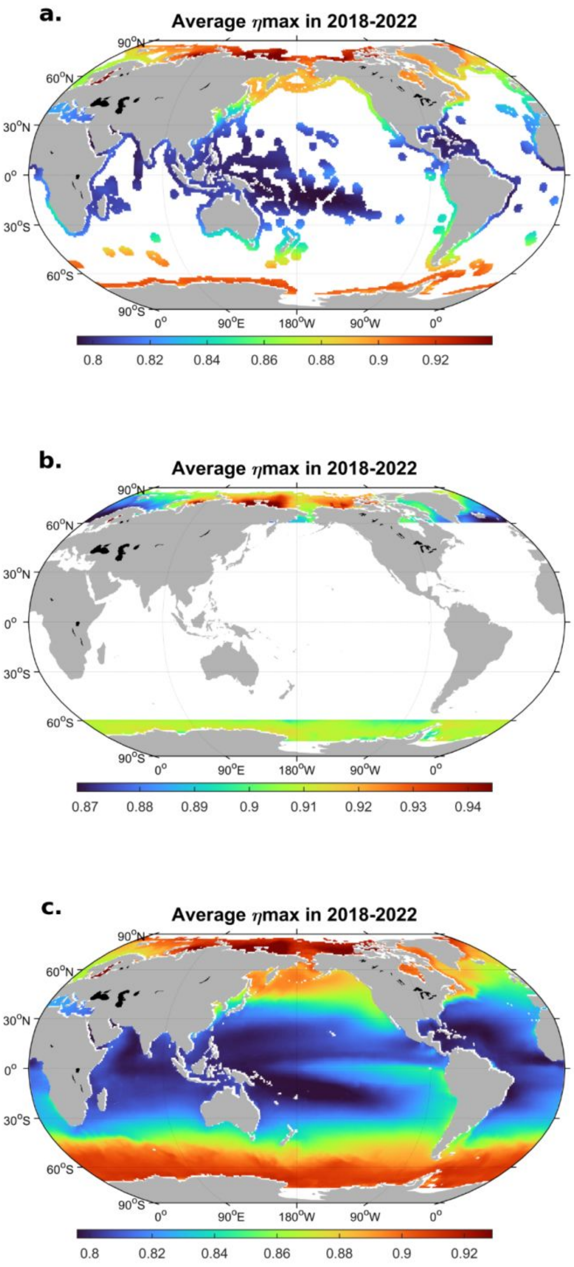

Fig. S5: Average ηmax for coastal, polar and global regions. Maps showing the 2018-2022 ηmax values in the grid cells averaged for three different regions: a) "coastal", using grid cells within 300 km of the nearest coast; b) "polar" using grid cells north of 60°N or south of 60°S and c) "global" using all surface ocean grid cells.

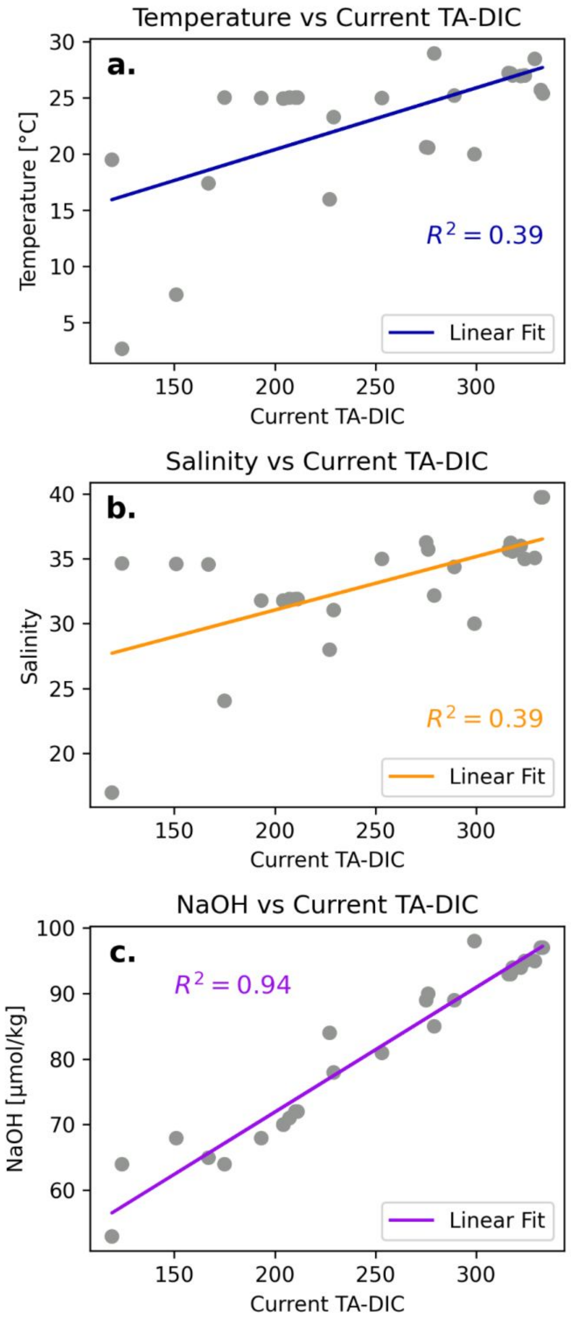

Fig. S6: Correlation of current TA-DIC vs temperature, salinity and NaOH addition . Current conditions TA-DIC for each species vs a) temperature in °C, b) salinity, and c) NaOH addition in µmol kg^{-1} required to restore pre-industrial TA-DIC. Grey dots show the experimental data for each species. Temperature and salinity were averaged per species.

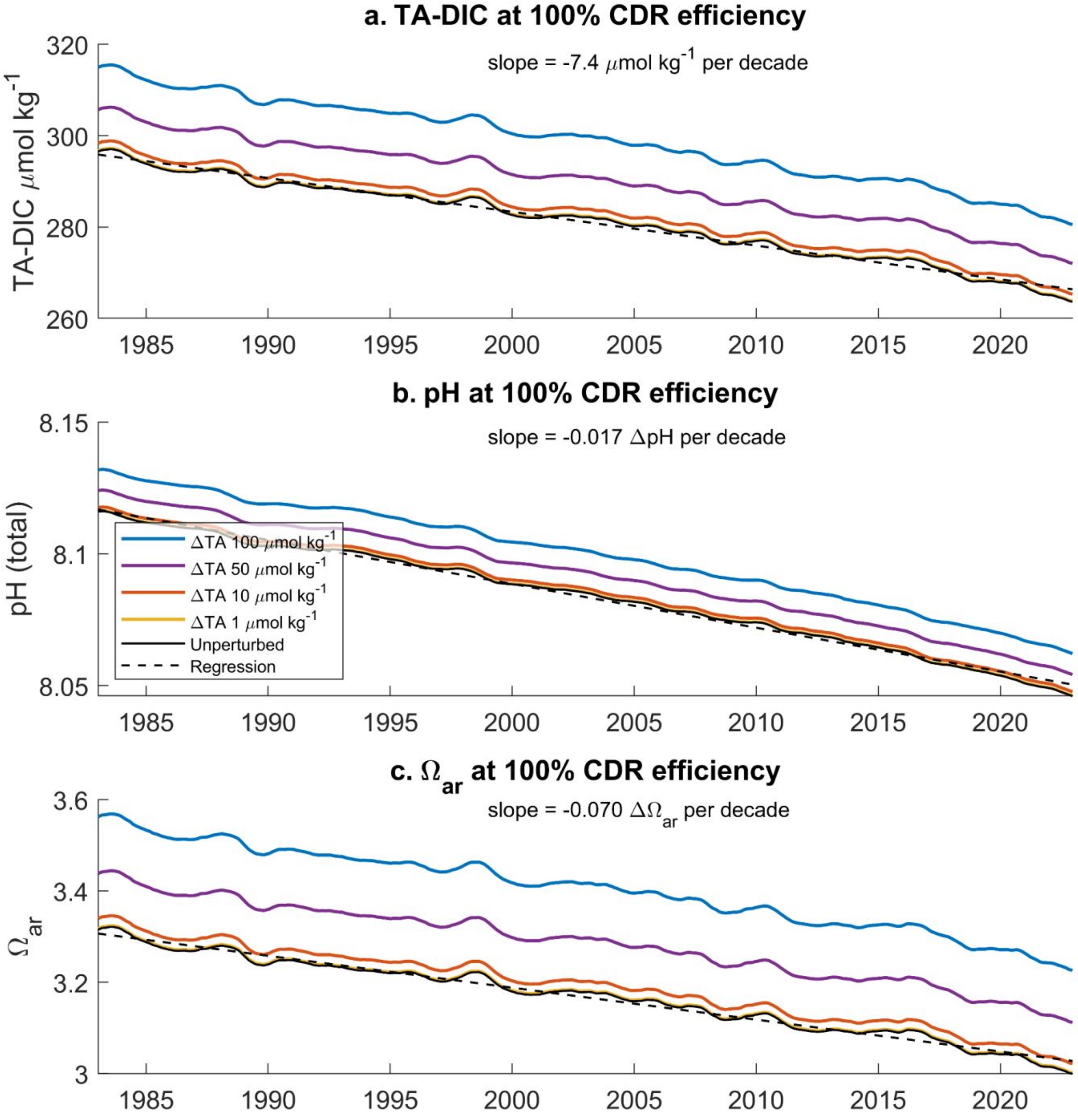

Fig. S7: Coastal region TA-DIC, pH_T and Ω_{ar} response to TA addition over time for 100% CDR efficiency. Differences in a) TA-DIC, b) pH_T and c) Ω_{ar} at different TA additions from 1985-2022 compared to the control assuming ηmax = 0.832 and 100% CDR efficiency.

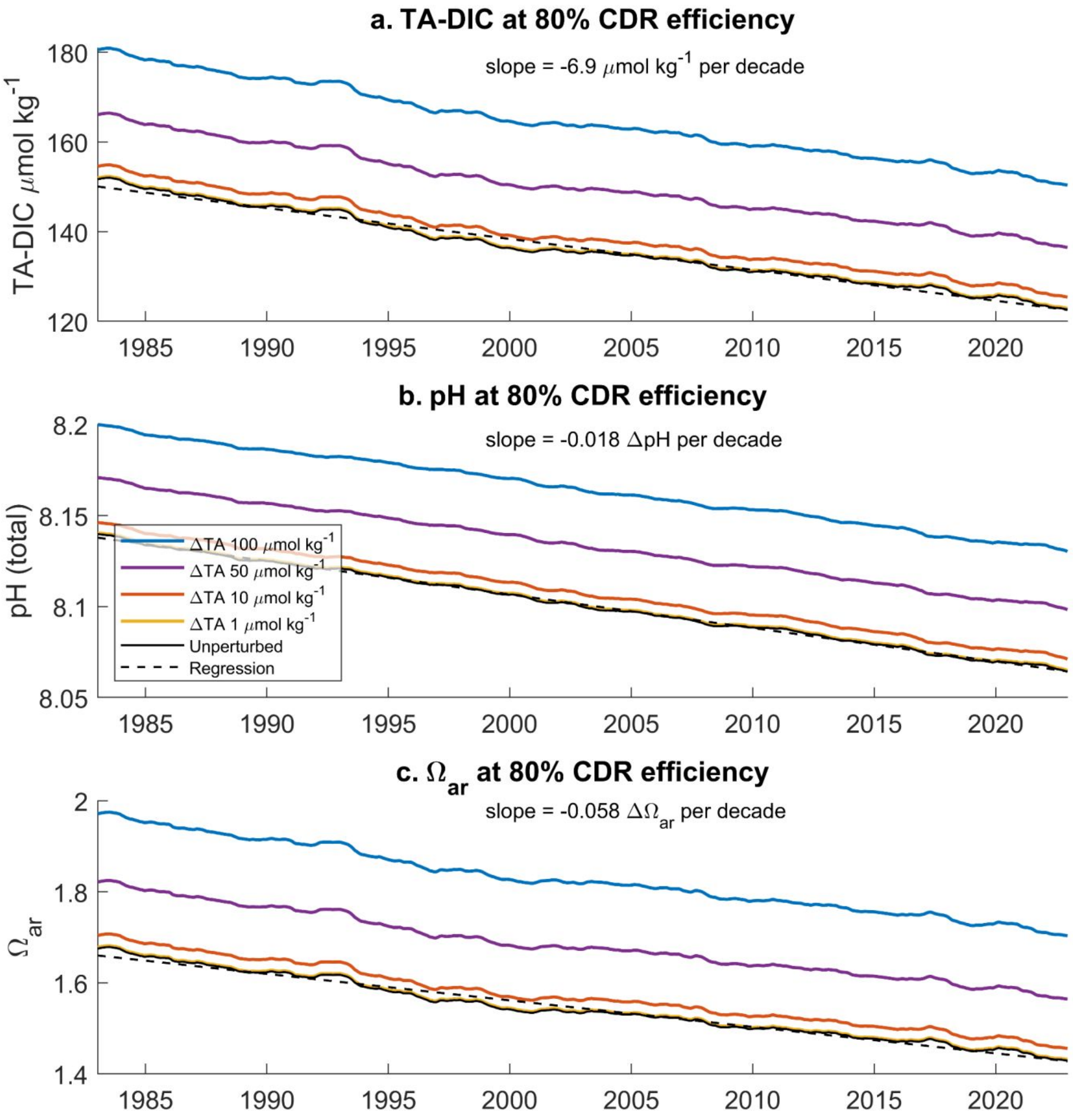

Fig. S8: Polar region TA-DIC, pH_T and Ω_{ar} response to TA addition over time for 80% CDR efficiency. Differences in a) TA-DIC, b) pH_T and c) Ω_{ar} at different TA additions from 1985-2022 compared to the control assuming ηmax = 0.904 and 80% CDR efficiency.

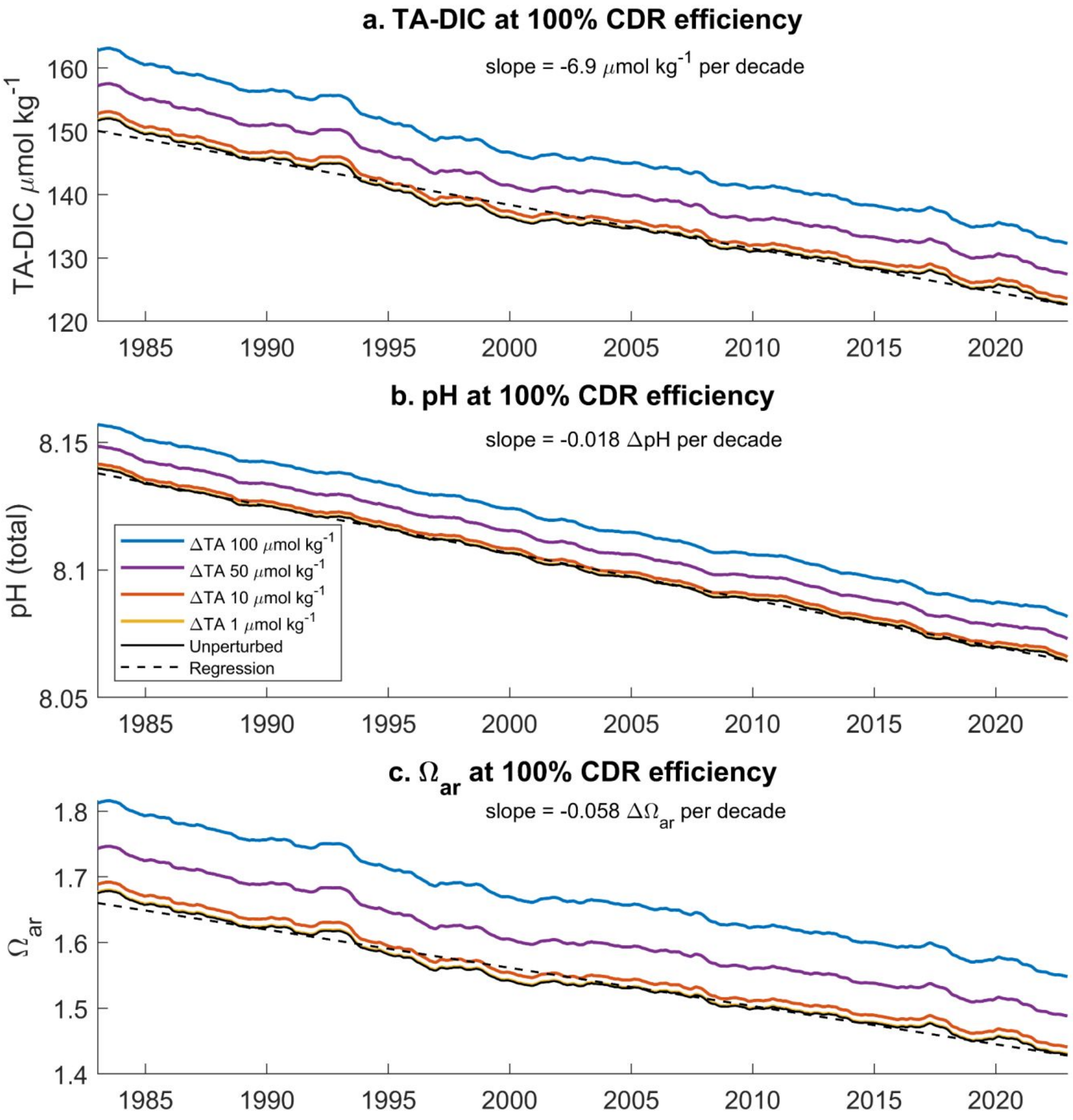

Fig. S9: Polar region TA-DIC, pH_T and Ω_{ar} response to TA addition over time for 100% CDR efficiency. Differences in a) TA-DIC, b) pH_T and c) Ω_{ar} at different TA additions from 1985-2022 compared to the control assuming ηmax = 0.904 and 80% CDR efficiency.

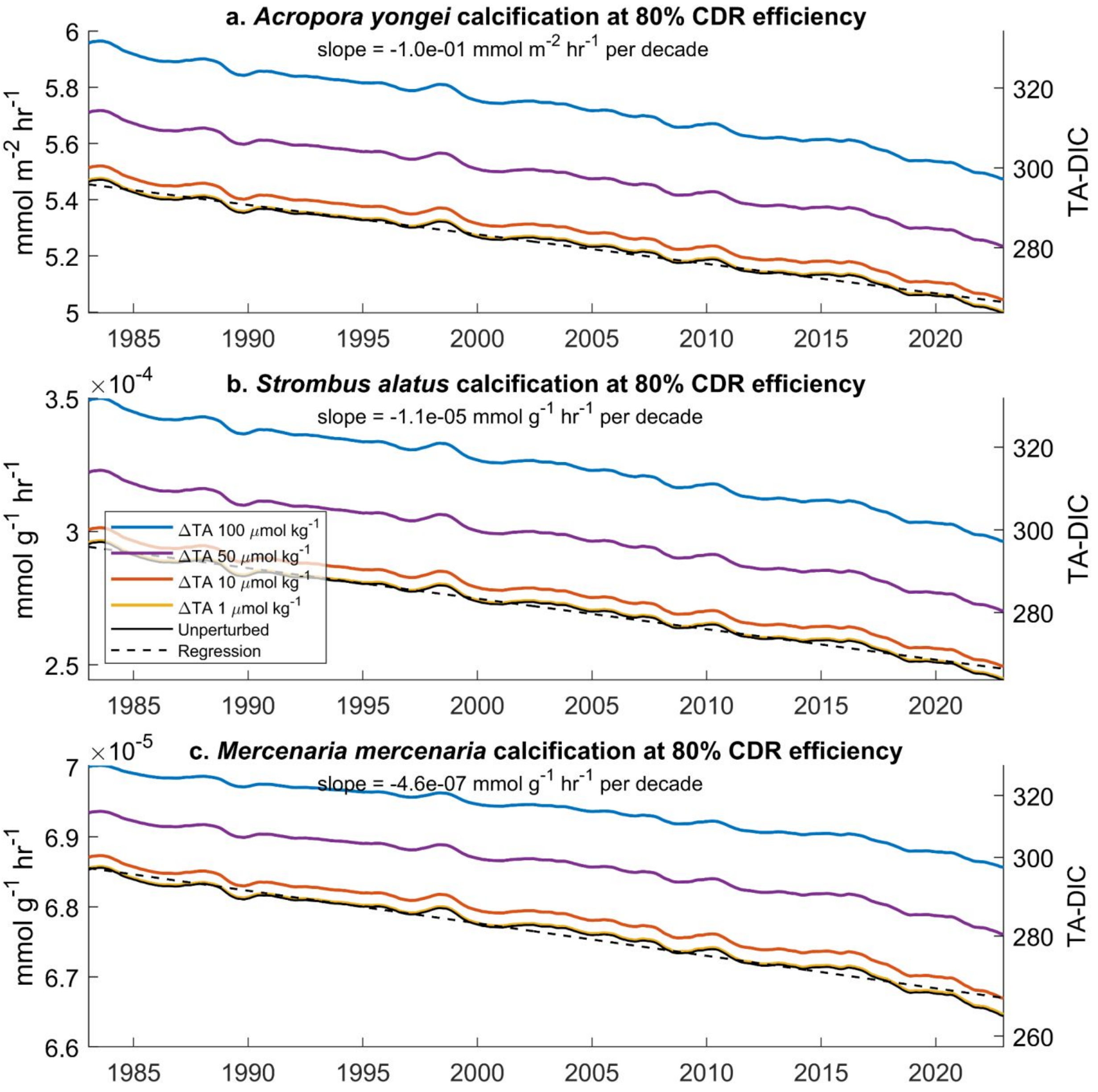

Fig. S10: Three species' calcification rate response to TA addition over time for 80% CDR efficiency. TA-DIC and related calcification rates of a) *Acropora yongei*, b) *Strombus alatus* and c) *Mercenaria mercenaria*) at different TA additions over the 1985-2022 period, with ηmax = 0.832 for the coastal region and 80% CDR efficiency. The dotted black line and slope is the linear regression of the unperturbed scenario.

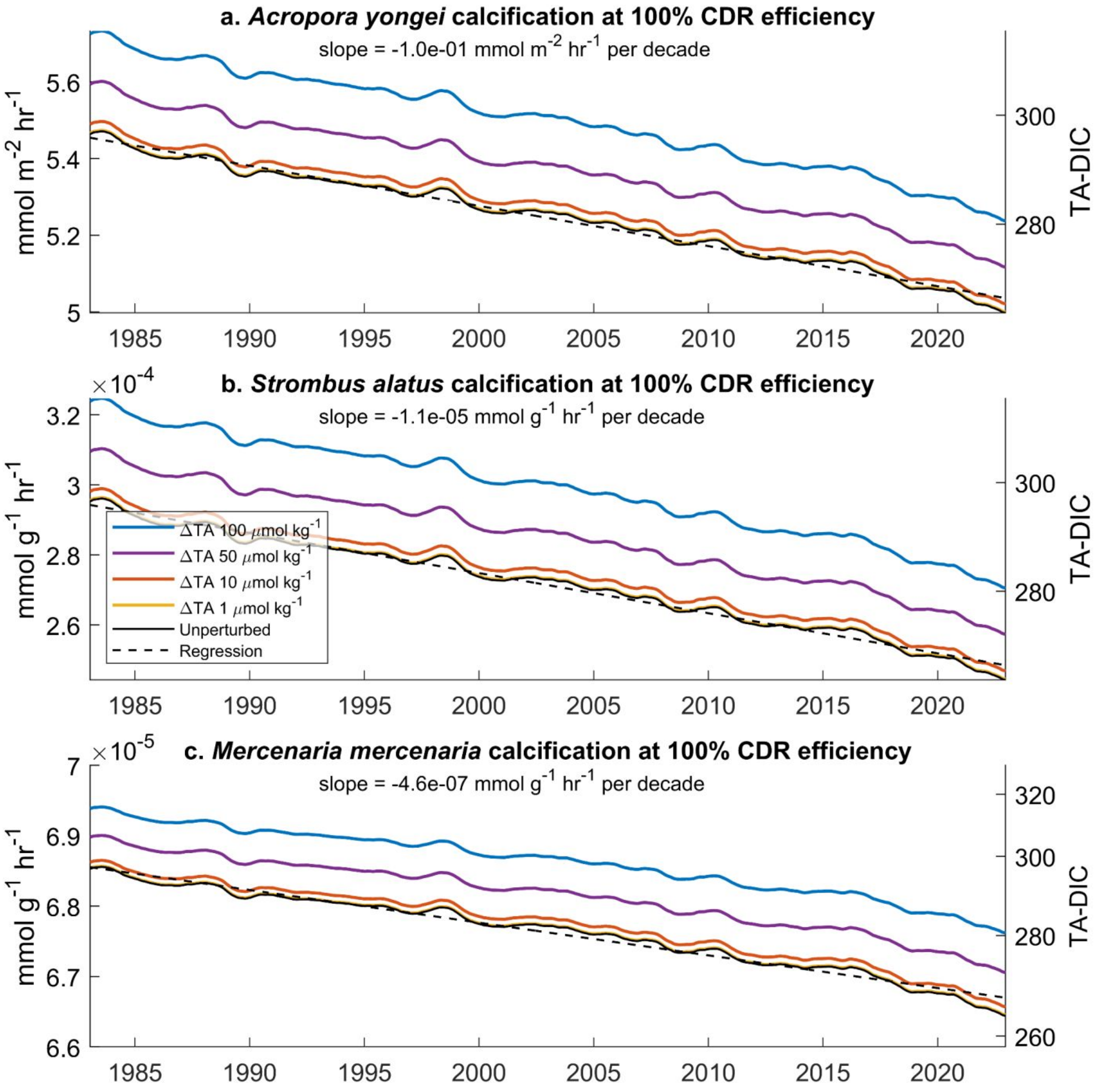

Fig. S11: Three species' calcification rate response to TA addition over time for 100% CDR efficiency. TA-DIC and related calcification rates of a) *Acropora yongei*, b) *Strombus alatus* and c) *Mercenaria mercenaria*) at different TA additions over the 1985-2022 period, with ηmax = 0.832 for the coastal region and 100% CDR efficiency. The dotted black line and slope is the linear regression of the unperturbed scenario.

Table S1: Mean ηmax for coastal, polar and global regions. Shows the 2018-2022 ηmax values in the grid cells averaged for three different regions: a) "coastal", using grid cells within 300 km of the nearest coast; b) "polar" using grid cells north of 60°N or south of 60°S and c) "global" using all surface ocean grid cells.

**Region**	**Mean η_{max}**	**Standard deviation η_{max}**
Coastal	0.832	0.0404
Polar	0.904	0.0163
Global	0.837	0.0377

Table S2: Collection locations and related ηmax regions per species. Collection locations per species and the associated regions (coastal, polar or global) used to determine regional ηmax values.

Studies	Group	Species	Collection location	Coast/Polar
(87)	Algae	*Halimeda opuntia*	Great Barrier Reef	Coastal
(88)	Algae	*Hydrolithon reinboldii*	Moorea, French Polynesia	Coastal
(89)	Algae	*Lithophyllum sp.*	Bocas del Toro Archipelaho, Caribbean	Coastal
(90)	Algae	*Porolithon onkodes*	Cooks Bay Moorea	Coastal
(91, 92)	Algae	*Sporolithon durum*	Salmon Bay, Rottnest Island, Western Australia	Coastal
(91, 92)	Coral	*Acropora yongei*	Salmon Bay, Rottnest Island, Western Australia	Coastal
(58)	Coral	*Duncanopsammia axifuga*	Pacific: Northern Australia and South China Sea	Coastal
(58)	Coral	*Montastraea cavernosa*	Caribbean	Coastal
(88)	Coral	*Pavona cactus*	Moorea, French Polynesia	Coastal
(92)	Coral	*Plesiastrea versipora*	Salmon Bay, Rottnest Island, Western Australia	Coastal
(94)	Coral	*Pocillopora verrucosa*	Moorea, French Polynesia	Coastal
(88)	Coral	*Porites rus*	Moorea, French Polynesia	Coastal
(83)	Coral	*Siderastrea radians*	Florida Bay	Coastal
(83)	Coral	*Solenastrea hyades*	Florida Bay	Coastal
(96)	Echino.	*Eucidaris tribuloides*	Nearshore Atlantic	Coastal
(98)	Foram.	*Marginopora vertebralis*	Papua New Guinea & Great Barrier Reef	Coastal
(82)	Gastro.	*Concholepas concholepas*	Intertidal southern Chile	Coastal
(96)	Gastro.	*Littorina littorea*	Intertidal Atlantic	Coastal
(96)	Gastro.	*Strombus alatus*	Shallow, warm Atlantic	Coastal
(96)	Gastro.	*Urosalpinx cinerea*	Atlantic coast	Coastal
(96)	Mollusks	*Argopecten irradians*	Atlantic coast	Coastal
(99)	Mollusks	*Crassostrea gigas*	Eastern Scheldt Estuary	Coastal
(81, 96)	Mollusks	*Crassostrea virginica*	Atlantic coast/Chesapeake Bay	Coastal
(96)	Mollusks	*Mercenaria mercenaria*	Atlantic coast	Coastal
(96)	Mollusks	*Mya arenaria*	Atlantic coast	Coastal

(66, 96)	Mollusks	*Mytilus edulis*	Atlantic coast/Western Scheldt Estuary	Coastal
(37)	Ptero.	*Limacina helicina*	Spitsbergen	Polar

Table S3: Additional information Table 1. This table gives the study type for each species' (combined) experimental dataset as well as the number of datapoints. It also gives statistical metrics for each species' regression model, as well as the TA and DIC concentrations for the current and pre-industrial conditions.

									Experimental conditions		Current conditions		Pre-industrial conditions	
**Species**	**Study type**	***n***	**Slope**	**Intercept**	**Exp.**	**p**	**R²**	**RMSE**	**T [°C]**	**S**	**TA [µmol kg⁻¹]**	**DIC [µmol kg⁻¹]**	**TA [µmol kg⁻¹]**	**DIC [µmol kg⁻¹]**
Halimeda opuntia	Lab	*24*	1.71E-04	3.32E-02		0.009	0.27	2.22E-02	25.0 - 25.4	34.4 - 34.4	2276	1984	2276	1892
Hydrolithon reinboldii	Lab	*72*	7.48E-06	9.80E-04		0.005	0.11	2.60E-03	26.9 - 27.1	36.0 - 36.0	2235	1993	2235	1935
Lithophyllum sp.	Lab + field	*420*	-3.96E-01	2.44E-01	-1.28E-02	0	0.12	1.13E-01	28.8 - 29.2	32.2 - 32.2	2134	1841	2134	1748
Porolithon onkodes		*425*	1.66E-03	1.61E+00		0.001	0.03	8.09E-01	26.9 - 27.5	35.3 - 36.1	2310	1977	2310	1873
Sporolithon durum	Lab	*64*	9.02E-04	-8.52E-02		0.001	0.15	1.75E-01	20.4 - 20.9	35.2 - 36.3	2348	2090	2348	2008
Duncanopsammia axifuga	Lab	*27*	2.55E-02	1.16E+01		0.001	0.34	5.07E+00	20.4 - 20.9	35.2 - 36.3	2371	1885	2371	1606
Montastraea cavernosa	Lab	*65*	1.11E-03	1.64E+00		0.014	0.09	5.04E-01	27.0 - 27.0	35.0 - 35.0	2371	1885	2371	1606
Pavona cactus	Lab	*72*	3.54E-03	2.48E+00		0	0.18	9.32E-01	27.0 - 27.0	35.0 - 35.0	2291	1963	2291	1863
Plesiastrea versipora	Lab	*49*	2.66E-03	1.61E+00		0.007	0.15	6.00E-01	27.1 - 27.3	36.1 - 36.4	2358	2088	2358	2001
Pocillopora verrucosa	Lab	*60*	3.20E-03	1.46E+00		0.013	0.1	8.30E-01	20.5 - 20.9	36.3 - 36.3	2321	2000	2321	1904
Porites rus	Lab	*72*	6.28E-03	4.25E+00		0.002	0.13	2.03E+00	26.9 - 27.0	35.6 - 35.7	2291	1963	2291	1863
Siderastrea radians	Field	*75*	1.68E-02	-1.50E+00		0	0.17	2.77E+00	27.1 - 27.3	36.1 - 36.4	2420	2030	2420	1891
Solenastrea hyades	Field	*64*	-2.55E+01	2.84E+00	-1.70E-02	0	0.23	2.03E+00	20.0 - 30.8	32.2 - 47.3	2424	2029	2424	1887
Amphibalanus improvisus	Lab	*36*	5.32E-06	8.31E-04		0	0.43	4.00E-04	20.0 - 30.8	32.4 - 47.2	1608	1643	1608	1636
Eucidaris tribuloides	Lab	*18*	-1.17E-03	6.32E-04	-1.60E-02	0	0.84	4.00E-04	24.9 - 25.1	31.7 - 31.9	1744	1563	1744	1500
Marginopora vertebralis	Lab	*47*	-2.64E-02	1.90E-03	-3.52E-02	0	0.4	4.00E-04	28.5 - 28.5	35.0 - 35.2	2332	1927	2332	1771
Concholepas concholepas	Lab	*74*	-2.27E-03	3.39E-03	-4.50E-02	0	0.29	9.00E-04	15.6 - 19.3	34.5 - 34.6	1788	1682	1788	1647

Littorina littorea	Lab	*42*	1.45E-06	-7.34E-05		0	0.33	2.00E-04	24.9 - 25.1	31.7 - 31.9	1770	1568	1770	1492
Strombus alatus	Lab	*21*	1.55E-06	-1.65E-04		0	0.66	1.00E-04	24.9 - 25.1	31.7 - 31.9	1770	1568	1770	1492
Urosalpinx cinerea	Lab	*33*	1.21E-06	-9.46E-05		0	0.56	1.00E-04	24.9 - 25.1	31.7 - 31.9	1770	1568	1770	1492
Argopecten irradians	Lab	*18*	1.33E-06	1.80E-04		0.009	0.35	2.00E-04	25.0 - 25.1	31.7 - 32.1	1833	1598	1833	1499
Crassostrea gigas	Lab	*20*	7.48E-07	1.19E-04		0	0.64	0.00E+00	20.0 - 20.0	30.0 - 30.0	2453	2206	2453	2128
Crassostrea virginica	Lab	*28*	-1.05E-04	2.51E-04	-1.33E-02	0	0.51	3.00E-04	20.0 - 32.0	16.0 - 32.1	1868	1770	1868	1748
Mercenaria mercenaria	Lab	*25*	-1.53E-04	7.28E-05	-1.20E-02	0	0.83	0.00E+00	25.0 - 25.1	31.7 - 32.1	1833	1598	1833	1499
Mya arenaria	Lab	*14*	7.18E-06	-6.53E-04		0	0.71	3.00E-04	25.0 - 25.1	31.7 - 32.1	1833	1598	1833	1499
Mytilus edulis	Lab	*86*	6.20E-07	1.57E-04		0.008	0.08	2.00E-04	20.0 - 25.1	30.0 - 32.1	2127	1884	2127	1792
Limacina helicina	Lab	*12*	3.19E-06	1.95E-05		0	0.86	1.00E-04	0.5 - 5.0	34.5 - 34.8	2283	2118	2283	2015

Table S4: Na_2CO_3 addition required to restore pre-industrial conditions per species. Summary of all OA studies for linear and threshold positive responders from which the chemical and biological data was collected, including species/group names and calcification rate units. As opposed to Table 1 where TA is added in the form of NaOH, this table shows the Na_2CO_3 addition required to return to TA-DIC from current to pre-industrial conditions and the calcification rate increase upon 10 μmol kg^{-1} Na_2CO_3 addition, considering both non-equilibrated and equilibrated OAE.

					OAE (to restore PI conditions)		OAE (calc. increase +50 μmol kg^{-1} Na_2CO_3)	
**Studies**	**Data points**	**Group**	**Species**	**Rate unit**	**ΔNa_2CO_3, no eq. [μmol kg^{-1}]**	**ΔNa_2CO_3, with eq. [μmol kg^{-1}]**	**No eq. [%]**	**With eq. [%]**
(87)	24	Algae	*Halimeda opuntia*	mmol/m²/hr	183	390	5.14	2.41
(88)	72	Algae	*Hydrolithon reinboldii*	mmol/g/hr	115	245	6.69	3.14
(89)	420	Algae	*Lithophyllum sp.*	mmol/g/hr	186	397	1.08	0.55
(90)	425	Algae	*Porolithon onkodes*	mmol/m²/hr	208	445	1.92	0.90
(91, 92)	64	Algae	*Sporolithon durum*	mmol/m²/hr	163	348	15.24	7.15
(91, 92)	81	Coral	*Acropora yongei*	mmol/m²/hr	163	348	7.20	3.38
(58)	27	Coral	*Duncanopsammia axifuga*	mmol/m²/hr	559	1191	2.66	1.25
(58)	65	Coral	*Montastraea cavernosa*	mmol/m²/hr	559	1191	1.27	0.60
(88)	72	Coral	*Pavona cactus*	mmol/m²/hr	200	427	2.43	1.14
(92)	49	Coral	*Plesiastrea versipora*	mmol/m²/hr	173	369	2.86	1.34
(94)	60	Coral	*Pocillopora verrucosa*	mmol/m²/hr	192	409	3.22	1.51
(88)	72	Coral	*Porites rus*	mmol/m²/hr	200	426	2.49	1.17
(83)	75	Coral	*Siderastrea radians*	mmol/m²/hr	276	590	8.30	3.90
(83)	64	Coral	*Solenastrea hyades*	mmol/m²/hr	283	603	0.37	0.20
(96)	18	Echino.	*Eucidaris tribuloides*	mmol/g/hr	126	269	3.76	1.95

(98)	47	Foram.	*Marginopora vertebralis*	mmol/g/hr	312	665	0.00	0.00
(82)	74	Gastro.	*Concholepas concholepas*	mmol/g/hr	69	147	0.37	0.23
(96)	42	Gastro.	*Littorina littorea*	mmol/g/hr	153	326	16.50	7.75
(96)	21	Gastro.	*Strombus alatus*	mmol/g/hr	153	326	26.09	12.25
(96)	33	Gastro.	*Urosalpinx cinerea*	mmol/g/hr	153	326	20.19	9.48
(96)	18	Mollusk	*Argopecten irradians*	mmol/g/hr	198	422	6.75	3.17
(99)	20	Mollusk	*Crassostrea gigas*	mmol/g/hr	156	333	6.17	2.90
(81, 96)	28	Mollusk	*Crassostrea virginica*	mmol/g/hr	45	95	3.67	1.87
(96)	25	Mollusk	*Mercenaria mercenaria*	mmol/g/hr	198	422	3.68	1.86
(96)	14	Mollusk	*Mya arenaria*	mmol/g/hr	198	422	17.35	8.15
(66, 96)	86	Mollusk	*Mytilus edulis*	mmol/g/hr	184	392	5.04	2.37
(37)	12	Ptero.	*Limacina helicina*	mmol/g/hr	206	584	14.65	5.18

Table S5: Time mitigations for TA-DIC, pH and Ω_{ar}. For the three variables TA-DIC, pH and Ω_{ar}, we give the slopes, mean change and year shifts for 1, 10, 50 and 100 µmol kg^{-1} NaOH addition (see Fig. S7-S10) for the coastal and polar regions, as well as 80% and 100% CDR efficiency.

Region	CDR efficiency	Variable	Mean control	Slope of unperturbed per decade	Mean Δ(Variable) ΔTA =1 µmol kg^{-1}	Mean Δ(Variable) ΔTA =10 µmol kg^{-1}	Mean Δ(Variable) ΔTA =50 µmol kg^{-1}	Mean Δ(Variable) ΔTA =100 µmol kg^{-1}	Year shift ΔTA =1 µmol kg^{-1}	Year shift ΔTA =10 µmol kg^{-1}	Year shift ΔTA =50 µmol kg^{-1}	Year shift ΔTA =100 µmol kg^{-1}
Coastal	80%	TA-DIC	2.81E+02	-7.39E+00	3.41E-01	3.41E+00	1.71E+01	3.42E+01	-0.46	-4.61	-23.1	-46.3
		pH_T	8.08E+00	-1.66E-02	4.80E-04	4.78E-03	2.34E-02	4.55E-02	-0.29	-2.87	-14	-27.3
		Ω_{ar}	3.17E+00	-6.99E-02	3.97E-03	3.97E-02	2.00E-01	4.03E-01	-0.57	-5.68	-28.6	-57.6
	100%	TA-DIC	2.81E+02	-7.39E+00	1.76E-01	1.76E+00	8.82E+00	1.77E+01	-0.24	-2.38	-11.9	-24
		pH_T	8.08E+00	-1.66E-02	1.64E-04	1.63E-03	8.10E-03	1.60E-02	-0.1	-0.98	-4.9	-9.6
		Ω_{ar}	3.17E+00	-6.99E-02	2.35E-03	2.35E-02	1.18E-01	2.38E-01	-0.34	-3.36	-16.9	-34
Polar	80%	TA-DIC	1.36E+02	-6.89E+00	2.82E-01	2.82E+00	1.41E+01	2.83E+01	-0.41	-4.09	-20.5	-41
		pH_T	8.10E+00	-1.84E-02	6.78E-04	6.73E-03	3.28E-02	6.35E-02	-0.37	-3.66	-17.8	-34.5
		Ω_{ar}	1.54E+00	-5.82E-02	2.77E-03	2.78E-02	1.41E-01	2.85E-01	-0.48	-4.78	-24.2	-49
	100%	TA-DIC	1.36E+02	-6.89E+00	1.02E-01	1.02E+00	5.13E+00	1.03E+01	-0.15	-1.48	-7.4	-15
		pH_T	8.10E+00	-1.84E-02	1.78E-04	1.78E-03	8.82E-03	1.74E-02	-0.1	-0.97	-4.8	-9.5
		Ω_{ar}	1.54E+00	-5.82E-02	1.26E-03	1.26E-02	6.33E-02	1.28E-01	-0.22	-2.16	-10.9	-21.9

Table S6: Time mitigation for calcification rate for all species at 80% CDR efficiency. TA-DIC and related calcification rates at different TA additions over the 1985-2022 period, with ηmax = 0.832 for all species living in the coastal region and ηmax = 0.904 for *Limacina helicina* in the polar region, for 80% CDR efficiency. The RPD slope is the percent change in the unperturbed calcification rate per decade. The RPD ΔCalc. is the difference between perturbed vs unperturbed calcification rates divided by the mean unperturbed calcification rate (relative % difference). The year shift represents the difference between the perturbed and unperturbed calcification rates divided by the slope of unperturbed calcification rate.

**Group**	**Species**	**RPD slope**	**RPD ΔCalc. [%] for ΔTA =10 µmol kg^{-1}**	**RPD ΔCalc. [%] for ΔTA = 50 µmol kg^{-1}**	**RPD ΔCalc. [%] for ΔTA =100 µmol kg^{-1}**	**Year shift ΔTA =10 µmol kg^{-1}**	**Year shift ΔTA =50 µmol kg^{-1}**	**Year shift ΔTA =100 µmol kg^{-1}**
Algae	*Halimeda opuntia*	-1.6	0.7	3.6	7.2	-4.61	-23.1	-46.2
Algae	*Hydrolithon reinboldii*	-1.8	0.8	4.1	8.3	-4.61	-23.1	-46.2
Algae	*Lithophyllum sp.*	-0.4	0.2	0.9	1.6	-4.49	-20.7	-37.3
Algae	*Porolithon onkodes*	-0.6	0.3	1.4	2.7	-4.61	-23.1	-46.2
Algae	*Sporolithon durum*	-4	1.8	9.1	18.3	-4.61	-23.1	-46.2
Coral	*Acropora yongei*	-2	0.9	4.6	9.2	-4.61	-23.1	-46.2
Coral	*Duncanopsammia axifuga*	-1	0.5	2.3	4.6	-4.61	-23.1	-46.2
Coral	*Montastraea cavernosa*	-0.4	0.2	1	1.9	-4.61	-23.1	-46.2
Coral	*Pavona cactus*	-0.8	0.3	1.7	3.5	-4.61	-23.1	-46.2
Coral	*Plesiastrea versipora*	-0.8	0.4	1.9	3.9	-4.61	-23.1	-46.2
Coral	*Pocillopora verrucosa*	-1	0.5	2.3	4.6	-4.61	-23.1	-46.2
Coral	*Porites rus*	-0.8	0.4	1.8	3.6	-4.61	-23.1	-46.2
Coral	*Siderastrea radians*	-3.8	1.8	8.9	17.8	-4.61	-23.1	-46.2
Coral	*Solenastrea hyades*	-1	0.5	2.1	3.6	-4.46	-19.9	-34.9
Echinoderm	*Eucidaris*	-0.3	0.1	0.5	0.9	-4.47	-20.1	-35.5

	tribuloides							
Foraminifera	*Marginopora vertebralis*	-0.02	0.01	0.03	0.05	-4.34	-17.4	-26.9
Gastropod	*Concholepas concholepas*	-0.0001	0	0.0001	0.0002	-4.29	-16.2	-23.8
Gastropod	*Littorina littorea*	-3.2	1.5	7.4	14.8	-4.61	-23.1	-46.2
Gastropod	*Strombus alatus*	-4.2	1.9	9.7	19.5	-4.61	-23.1	-46.2
Gastropod	*Urosalpinx cinerea*	-3.6	1.7	8.4	16.8	-4.61	-23.1	-46.2
Mollusks	*Argopecten irradians*	-1.8	0.8	4.1	8.2	-4.61	-23.1	-46.2
Mollusks	*Crassostrea gigas*	-1.7	0.8	3.9	7.8	-4.61	-23.1	-46.2
Mollusks	*Crassostrea virginica*	-0.1	0	0.2	0.4	-4.49	-20.6	-37
Mollusks	*Mercenaria mercenaria*	-0.7	0.3	1.4	2.6	-4.5	-20.8	-37.8
Mollusks	*Mya arenaria*	-3.9	1.8	9	18	-4.61	-23.1	-46.2
Mollusks	*Mytilus edulis*	-1.4	0.6	3.2	6.4	-4.61	-23.1	-46.2
Pteropod	*Limacina helicina*	-4.8	2	9.9	19.8	-4.09	-20.5	-41

Table S7: Time mitigation for calcification rate for all species at 100% CDR efficiency.. TA-DIC and related calcification rates at different TA additions over the 1985-2022 period, with ηmax = 0.832 for all species living in the coastal region and and ηmax = 0.904 for *Limacina helicina* in the polar region, for 100% CDR efficiency. RPD slope is the slope of unperturbed calcification rate divided by the mean unperturbed calcification rate (% change in calcification per decade). The RPD ΔCalc. is the difference between perturbed vs unperturbed calcification rates divided by the mean unperturbed calcification rate (relative % difference). The year shift represents the difference between the perturbed and unperturbed calcification rates divided by the slope of unperturbed calcification rate.

**Group**	**Species**	**RPD slope**	**RPD ΔCalc. [%] for ΔTA =10 µmol kg^{-1}**	**RPD ΔCalc. [%] for ΔTA =50 µmol kg^{-1}**	**RPD ΔCalc. [%] for ΔTA =100 µmol kg^{-1}**	**Year shift ΔTA =10 µmol kg^{-1}**	**Year shift ΔTA =50 µmol kg^{-1}**	**Year shift ΔTA =100 µmol kg^{-1}**
Algae	*Halimeda opuntia*	-1.6	0.4	1.9	3.7	-2.38	-11.9	-24
Algae	*Hydrolithon reinboldii*	-1.8	0.4	2.1	4.3	-2.38	-11.9	-24
Algae	*Lithophyllum sp.*	-0.4	0.1	0.5	0.9	-2.34	-11.2	-21.3
Algae	*Porolithon onkodes*	-0.6	0.1	0.7	1.4	-2.38	-11.9	-24
Algae	*Sporolithon durum*	-4	0.9	4.7	9.5	-2.38	-11.9	-24
Coral	*Acropora yongei*	-2	0.5	2.4	4.8	-2.38	-11.9	-24
Coral	*Duncanopsammia axifuga*	-1	0.2	1.2	2.4	-2.38	-11.9	-24
Coral	*Montastraea cavernosa*	-0.4	0.1	0.5	1	-2.38	-11.9	-24
Coral	*Pavona cactus*	-0.8	0.2	0.9	1.8	-2.38	-11.9	-24
Coral	*Plesiastrea versipora*	-0.8	0.2	1	2	-2.38	-11.9	-24
Coral	*Pocillopora verrucosa*	-1	0.2	1.2	2.4	-2.38	-11.9	-24
Coral	*Porites rus*	-0.8	0.2	0.9	1.9	-2.38	-11.9	-24
Coral	*Siderastrea radians*	-3.8	0.9	4.6	9.2	-2.38	-11.9	-24

Coral	*Solenastrea hyades*	-1	0.2	1.1	2.1	-2.33	-11	-20.6
Echinoderm	*Eucidaris tribuloides*	-0.3	0.1	0.3	0.5	-2.33	-11.1	-20.8
Foraminifera	*Marginopora vertebralis*	-0.02	0.004	0.02	0.03	-2.3	-10.2	-17.8
Gastropod	*Concholepas concholepas*	-0.0001	0.00002	0.0001	0.0001	-2.29	-9.9	-16.6
Gastropod	*Littorina littorea*	-3.2	0.8	3.8	7.7	-2.38	-11.9	-24
Gastropod	*Strombus alatus*	-4.2	1	5	10.1	-2.38	-11.9	-24
Gastropod	*Urosalpinx cinerea*	-3.6	0.9	4.3	8.7	-2.38	-11.9	-24
Mollusks	*Argopecten irradians*	-1.8	0.4	2.1	4.3	-2.38	-11.9	-24
Mollusks	*Crassostrea gigas*	-1.7	0.4	2	4	-2.38	-11.9	-24
Mollusks	*Crassostrea virginica*	-0.1	0	0.1	0.2	-2.34	-11.2	-21.3
Mollusks	*Mercenaria mercenaria*	-0.7	0.2	0.8	1.5	-2.34	-11.3	-21.5
Mollusks	*Mya arenaria*	-3.9	0.9	4.6	9.3	-2.38	-11.9	-24
Mollusks	*Mytilus edulis*	-1.4	0.3	1.7	3.3	-2.38	-11.9	-24
Pteropod	*Limacina helicina*	-4.8	0.7	3.6	7.2	-1.48	-7.4	-15
